# Characterization and Demonstration of Mock Communities as Control Reagents for Accurate Human Microbiome Community Measurements

**DOI:** 10.1128/spectrum.01915-21

**Published:** 2022-03-02

**Authors:** Dieter M. Tourlousse, Koji Narita, Takamasa Miura, Akiko Ohashi, Masami Matsuda, Yoshifumi Ohyama, Mamiko Shimamura, Masataka Furukawa, Ken Kasahara, Keishi Kameyama, Sakae Saito, Maki Goto, Ritsuko Shimizu, Riko Mishima, Jiro Nakayama, Koji Hosomi, Jun Kunisawa, Jun Terauchi, Yuji Sekiguchi, Hiroko Kawasaki

**Affiliations:** a Biomedical Research Institute, National Institute of Advanced Industrial Science and Technology (AIST), Tsukuba, Ibaraki, Japan; b Japan Microbiome Consortium (JMBC), Osaka, Japan; c Chitose Laboratory Corp., Kawasaki, Kanagawa, Japan; d Biological Resource Center, National Institute of Technology and Evaluation (NITE), Kisarazu, Chiba, Japan; e Institute of Food Sciences and Technologies, Ajinomoto Co., Inc., Kawasaki, Kanagawa, Japan; f Tohoku Medical Megabank Organization, Tohoku University, Aoba-ku, Sendai, Miyagi, Japan; g Department of Bioscience and Biotechnology, Faculty of Agriculture, Graduate School, Kyushu University, Nishi-ku, Fukuoka, Japan; h Laboratory of Vaccine Materials, Center for Vaccine and Adjuvant Research, and Laboratory of Gut Environmental System, National Institutes of Biomedical Innovation, Health and Nutrition (NIBIOHN), Ibaraki, Osaka, Japan; i Ono Pharmaceutical Co., Ltd., Osaka, Japan; University of Illinois at Urbana Champaign

**Keywords:** control reagents, human microbiome, metagenomics, standards

## Abstract

Standardization and quality assurance of microbiome community analysis by high-throughput DNA sequencing require widely accessible and well-characterized reference materials. Here, we report on newly developed DNA and whole-cell mock communities to serve as control reagents for human gut microbiota measurements by shotgun metagenomics and 16S rRNA gene amplicon sequencing. The mock communities were formulated as near-even blends of up to 20 bacterial species prevalent in the human gut, span a wide range of genomic guanine-cytosine (GC) contents, and include multiple strains with Gram-positive type cell walls. Through a collaborative study, we carefully characterized the mock communities by shotgun metagenomics, using previously developed standardized protocols for DNA extraction and sequencing library construction. Further, we validated fitness of the mock communities for revealing technically meaningful differences among protocols for DNA extraction and metagenome/16S rRNA gene amplicon library construction. Finally, we used the mock communities to reveal varying performance of metagenome-based taxonomic profilers and the impact of trimming and filtering of sequencing reads on observed species profiles. The latter showed that aggressive preprocessing of reads may result in substantial GC-dependent bias and should thus be carefully evaluated to minimize unintended effects on species abundances. Taken together, the mock communities are expected to support a myriad of applications that rely on well-characterized control reagents, ranging from evaluation and optimization of methods to assessment of reproducibility in interlaboratory studies and routine quality control.

**IMPORTANCE** Application of high-throughput DNA sequencing has greatly accelerated human microbiome research and its translation into new therapeutic and diagnostic capabilities. Microbiome community analyses results can, however, vary considerably across studies or laboratories, and establishment of measurement standards to improve accuracy and reproducibility has become a priority. The here-developed mock communities, which are available from the NITE Biological Resource Center (NBRC) at the National Institute of Technology and Evaluation (NITE, Japan), provide well-characterized control reagents that allow users to judge the accuracy of their measurement results. Widespread and consistent adoption of the mock communities will improve reproducibility and comparability of microbiome community analyses, thereby supporting and accelerating human microbiome research and development.

## INTRODUCTION

In the last decade, research into the human microbiome has greatly benefited from widespread access to high-throughput DNA sequencing as a powerful tool for interrogating complex microbial ecosystems, such as those found in the human gastrointestinal tract. This has enabled large-scale cohort studies that revealed numerous correlations between the taxonomic and functional composition of human-associated microbiota and host health (1, 2). As the mechanistic underpinnings of disease-causing alterations in the human microbiome are now starting to be better understood (3, 4), microbiome science is anticipated to lead to a myriad of new therapeutic and diagnostic applications in coming years (5–7). With this has also come an increased emphasis on quality assurance and standardization of methods for microbiome community analysis (8–10).

Reference materials form the basis of quality assurance and standardization across analytical disciplines. In the microbiome field, reference materials have generally taken the form of defined mixtures of genomic DNA or whole cells from several to a few tens of distinct microorganisms. Such mock communities have been used to characterize protocol-dependent biases and optimize or validate methods (11–15), to assess interlaboratory reproducibility in collaborative studies (16–18), and to evaluate run-to-run variability (19). In addition to mock communities, human whole stool samples have also been employed in several of the above studies and their development as reference materials is ongoing (20, 21).

Mock communities with known composition, in contrast to whole stool samples, are intended to provide a ‘ground truth’ to which measurement results can be compared in order to assess accuracy (11). To ensure fit for purpose, this requires careful characterization of the mock communities with respect to their purity and quantitative composition. Analysis of new mock communities should thus ideally be performed using multiple well-established orthogonal methods, preferably across multiple laboratories. Further, complete genome sequences should be available for all strains in order to facilitate interpretation of mock community measurement results.

Here, we report on the development and evaluation of mock communities intended to serve as control reagents for human microbiome community measurements by high-throughput DNA sequencing. The mock communities consist mainly of bacteria that are prevalent in the human gut and were formulated as near-even mixtures of genomic DNA or whole cells of 20 (DNA mock) or 18 (cell mock) bacterial strains. As part of the development of the mock communities, complete genome sequences were newly generated for 12 strains. We carefully characterized the mock communities by shotgun metagenomics, using previously validated standard operating procedures (SOPs) for DNA extraction and metagenomic library construction (11), across multiple laboratories in a collaborative study. Further, we established their fitness to reveal variability in measurement results generated by different methods and/or by changing laboratories, covering protocols for DNA extraction and metagenome and 16S rRNA gene amplicon sequencing. Finally, we explored use of the mock communities to compare metagenome-based taxonomic profilers and to assess the impact of read trimming and filtering on observed species profiles. The here-described mock communities, which are available from the NITE Biological Resource Center (NBRC) at the National Institute of Technology and Evaluation (NITE, Japan), and demonstration of their utility in typical usage cases are expected to contribute to ongoing efforts to improve consistency of human microbiota measurements through better standardization and quality assurance.

## RESULTS AND DISCUSSION

**FIG 1:**
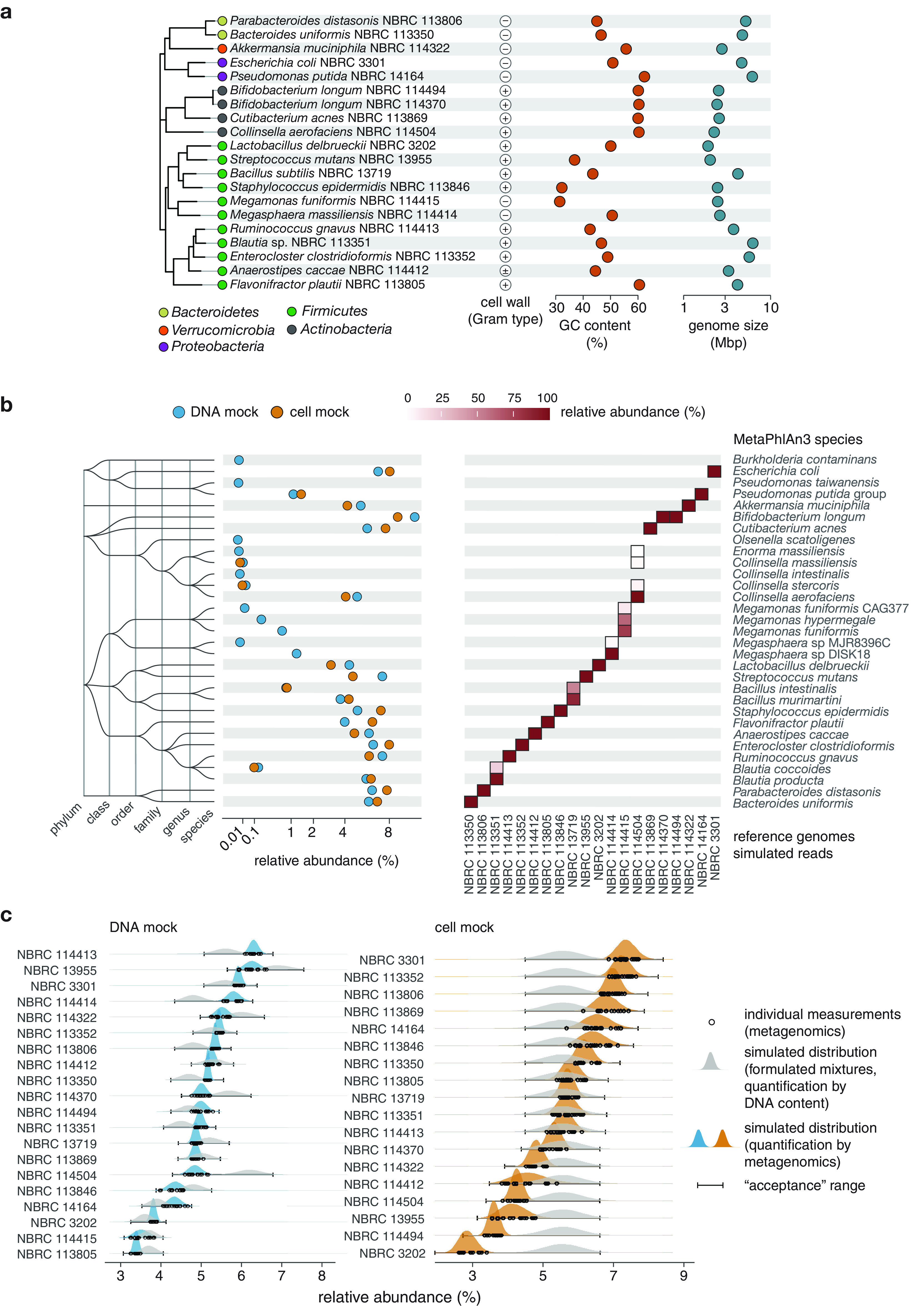
(a) Bacterial species included in the mock communities. The phylogenetic tree was inferred based on single-copy marker genes, using the GTDB-Tk. Phylum-level taxonomic assignments are indicated by colored circles shown at the leaves. Species features, that is Gram type, genomic GC content and genome size, are shown. (b) Characterization of the mock communities by shotgun metagenomics and taxonomic profiling with MetaPhlAn3. Species profiles were generated based on the combined sequencing data of all replicated measurements, performed following SOPs, for both mock communities (*n *= 16 and *n *= 20 for the DNA and cell mock community, respectively, covering multiple aliquots, SOPs and laboratories; Table S3 in the supplemental material). The tree represents a taxonomic dendrogram. The heatmap depicts species profiles of 10 million *in silico* generated reads (5 million 151-bp paired-end reads) for each of the genomes shown on the *x* axis, with fill colors showing estimated relative abundances as indicated in the legend. (c) Relative abundance of each strain in the mock communities as assigned based on total DNA content, during formulation, and as measured by shotgun metagenomics. Empty circles shown individual shotgun metagenomics measurement results as mentioned for panel b. Gray and blue/orange density plots show the distribution of simulated strain-wise abundances as indicated in the legend. ‘Acceptance’ ranges for strain-wise abundances are shown as error bars. Note that strains NBRC 114414 (*M. massiliensis*) and NBRC 114415 (*M. funiformis*) were not included in the cell mock community.

The mock communities consist mostly of bacteria that are prevalent in the human gastrointestinal tract, covering the phyla *Bacteroidetes*, *Actinobacteriota*, *Verrucomicrobiota*, *Firmicutes* and *Proteobacteria* (Table 1 and Fig. 1a). In addition, two species associated with human skin microbiota, namely, Staphylococcus epidermidis and Cutibacterium acnes subsp. *acnes*, were included, along with the GC-rich bacterium Pseudomonas putida.

**TABLE 1:** Bacterial species included in the mock communities

Species	Culture collection	Nucleotide accession	Genome size (bp)	GC content (%)	16S rRNA genes	Cell wall (Gram-type)^*a*^	Relative abundance in DNA mock^*b*^ (%)	Relative abundance in cell mock^*c*^ (%)
Bacteroides uniformis	NBRC 113350	AP019724 – AP019728	4,989,532	46.2	4	–	4.7	5.6
*Blautia* sp.	NBRC 113351	CP084061	6,247,046	46.7	5	+	4.5	5.6
*Enterocloster clostridioformis*	NBRC 113352	BJLB01000001 – BJLB01000002	5,687,315	48.9	5	+	5.3	5.6
Parabacteroides distasonis	NBRC 113806	AP019729	5,179,960	45.0	7	–	4.8	5.6
Bacillus subtilis subsp. *subtilis*	NBRC 13719	AP019714 – AP019715	4,295,305	43.3	10	+	5.2	5.6
Streptococcus mutans	NBRC 13955	AP019720	2,018,796	36.9	5	+	6.9	5.6
Pseudomonas putida	NBRC 14164	AP013070	6,156,701	62.3	7	–	3.9	5.6
Lactobacillus delbrueckii subsp. *delbrueckii*	NBRC 3202	AP019750	1,910,306	50.1	8	+	3.6	5.6
Escherichia coli	NBRC 3301	CP048439 – CP048440	4,755,096	50.8	7	–	5.6	5.6
Flavonifractor plautii	NBRC 113805	CP084007	4,277,038	60.4	3	+	3.7	5.6
Staphylococcus epidermidis	NBRC 113846	CP084008 – CP084011	2,520,735	32.2	6	+	4.8	5.6
Cutibacterium acnes subsp. *acnes*	NBRC 113869	CP084017	2,560,907	60.0	3	+	5.0	5.6
Bifidobacterium longum	NBRC 114370	CP084012 – CP084013	2,594,022	60.1	5	+	5.7	5.6
Anaerostipes caccae	NBRC 114412	CP084016	3,284,789	44.5	4	±	5.3	5.6
Ruminococcus gnavus	NBRC 114413	CP084014 – CP084015	3,757,469	42.5	5	+	5.6	5.6
Megasphaera massiliensis	NBRC 114414	CP084019	2,610,024	50.6	7	–	4.8	0^*b*^
Megamonas funiformis	NBRC 114415	CP084018	2,464,533	31.5	6	–	3.7	0^*b*^
Collinsella aerofaciens	NBRC 114504	CP084004 – CP084006	2,278,612	60.3	5	+	6.2	5.6
Bifidobacterium longum subsp. *longum*	NBRC 114494	CP084020 – CP084022	2,534,372	60.1	4	+	4.7	5.6
Akkermansia muciniphila	NBRC 114322	CP084201 – CP084202	2,788,458	55.7	3	–	6.0	5.6

a The symbols +, − and ± indicate strains with Gram-positive, Gram-negative and Gram-variable type cells walls, respectively.

b Relative abundances represent values assigned during formulation of the mock communities, based on quantification of the total DNA content of individual strains prior to mixing.

c *M. massiliensis* and *M. funiformis* were excluded from the cell mock community.

While based on their composition the mock communities are mainly intended for assessment of fecal microbiota measurements, they are expected to be more broadly applicable. Specifically, species capture a wide range of genomic GC contents (from 31.5% for Megamonas funiformis NBRC 114415 to 62.3% for P. putida NBRC 14164) and include a suite of strains with reported Gram-positive type cell walls (hereafter referred to as Gram-positives). Both genomic GC content and cell wall structure represent two important sources of bias in metagenomic analyses, and the mock communities thus provide appropriate reagents for challenging protocols.

As part of the development of the mock communities, we generated complete genome sequences for 12 strains lacking publicly available genomes (Table S1), by short- and long-read sequencing (see Materials and Methods for details). Next, to formulate the DNA mock community, we determined the total DNA concentration of genomic DNA stocks of each strain by fluorometry. The stocks were then combined to generate a mixture containing nearly equal genome copy numbers of each strain, resulting in a mock community with relative abundances ranging from 3.6% for Lactobacillus delbrueckii subsp. *delbrueckii* NBRC 3202 to 6.9% for Streptococcus mutans NBRC 13955 (Table 1). A similar approach was followed for the cell mock community, by measuring the total DNA content of whole cell stocks through acid-catalyzed release of adenine from whole cells. This technique was originally devised for determining the efficiency of microbial cell lysis and DNA recovery (22) and we previously demonstrated that it could provide a reliable basis for quantifying whole cell mock communities (11). Cell stocks of individual strains were then combined into a mixture containing equal amounts of total DNA, or cell equivalents assuming a constant genome copy number per cell, for each of the strains. Note that two strains, namely, Megasphaera massiliensis NBRC 114414 and M. funiformis NBRC 114415, were omitted from the cell mock community, such that each strain had a relative abundance of 5.6% (Table 1).

To characterize the mock communities and establish their fitness for purpose in typical use cases, we set up a collaborative study involving five laboratories (Fig. S1 and Table S2 in the supplemental material). Firstly, to assess purity and quantify strain-wise abundances using a technique orthogonal to total DNA quantification used during formulation (see above), three of the laboratories analyzed the mock communities by shotgun metagenomics, following our previously developed SOPs (11). Secondly, to establish fitness for purpose of the mock communities in revealing differences among protocols, two of the laboratories also evaluated non-SOPs for DNA extraction and metagenomics library construction. Further, all five laboratories performed 16S rRNA gene (V4 hypervariable region) amplicon sequencing, using both a standardized protocol (Illumina’s two-step tailed PCR protocol) or alternative custom methods (Table S2).

### Characterization of the mock communities by shotgun metagenomics.

Based on the collaborative study outlined above, a total of 16 and 20 metagenomics measurements were performed for the DNA and cell mock communities, respectively, following our previously developed SOPs (Table S3 in the supplemental material). In short, the SOP for DNA extraction (denoted as protocol N) uses the ISOSPIN Fecal DNA kit, employing three rounds of bead beating to ensure effective lysis of Gram-positives. For library construction, two SOPs were employed, namely, protocol B (QIAseq FX DNA Library kit) involving enzymatic DNA fragmentation, and protocol K (SMARTer ThruPLEX DNA-Seq kit) using physical DNA fragmentation by focused ultrasonication. Sequencing was performed on an Illumina NextSeq (Lab A, note that libraries prepared by Lab B were sequenced by Lab A as shown in Fig. S1) or HiSeq instrument (Lab C), generating 2 × 151 and 2 × 101 bp reads, respectively. Replicates covered three aliquots for each mock community (Lab A), protocol N for DNA extraction (Lab A and B), and protocols B and K for library construction (Lab A, B and C).

To assess purity of the mock communities, we analyzed the sequencing data by MetaPhlAn3 (23), a well-established tool for metagenome profiling based on marker genes. Inspection of individual sequencing libraries showed that species profiles were highly consistent across replicates, without indication of library-specific impurities (Fig. S2 in the supplemental material). The lack of contaminants was further verified through analysis of sequencing data combined across all available replicates for both mock communities, in order to allow detection of potentially lower abundance contaminants (Fig. 1b). More specifically, detected bacteria belonging to genera not part of the mock communities, including *Burkholderia* and *Olsenella*, were estimated to be of very low abundance (less than 0.001% in the combined sequencing reads). For other genera, namely, *Bacillus*, *Megamonas*, *Megasphaera* and *Collinsella*, unexpected species were concluded not to represent contaminants because they were also identified in the corresponding simulated reads, and thus presumed to reflect incorrect identifications by MetaPhlAn3. Taken together, these data verified that the formulated mock communities can, for most practical purposes, be considered as free of contaminants.

To determine the relative abundance of each strain, we used kallisto’s quantification workflow involving pseudo-mapping of paired reads to the reference genomes and estimation of read counts for each strain by expectation maximization (24). Firstly, nearly all reads could be assigned to the reference genomes, with mapping rates of, on average, 99% for both mock communities. This verified that the reference genomes were suitable for quantitative analyses. Secondly, variability in strain-wise abundances among aliquots, protocols (two SOPs were used for library construction, see above) and laboratories were small (Fig. S3 in the supplemental material), with a variation around the grand mean of all replicates of 2.7% (qmCV, see Materials and Methods for definition) and 5.7% for the DNA and cell mock community, respectively. Thirdly, estimated relative abundances for the different strains were in reasonably good agreement with values assigned during formulation based on total DNA quantification (Fig. 1c). For the DNA mock community, abundances of individual strains deviated 1.1-fold (gmAFD as calculated based on the mean of strain-wise abundances across replicates, see Materials and Methods for definition) from the assigned values. For the cell mock community, differences between assigned and measured values were slightly higher, with a gmAFD of 1.2-fold. Further, Gram-positives in the cell mock community had a total abundance of 63.5 ± 0.4% (mean and sd across replicates), in good agreement with the expected value of 67.2%.

Following the strategy that we described previously (11, also see Materials and Methods), we next calculated guidance values for allowable errors (that is, the acceptable level of deviation from the expected composition) by considering differences between strain-wise abundances assigned during formulation by total DNA quantification and the above metagenomics measurement results. These values, which are provided in Table S4 in the supplemental material, can be used to judge the accuracy of measurement results. Although a more rigorous and formal appraisal will be warranted toward achieving consensus on the acceptable level of errors (9), setting acceptance values for key performance parameters is a prerequisite for enabling meaningful use of reference materials.

As can be observed in Fig. 1c, agreement between relative abundances assigned during formulation and values measured by metagenomics was lower for a few strains. Here, expected values were calculated based on total DNA concentrations of DNA or cell stocks of each strain prior to mixing. Compared to digital PCR, total DNA measurements were used as they were presumed to be less sensitive to varying genome coverage, e.g., near the origin of replication in growing cells, which may lead to biased quantification depending on the genes targeted by digital PCR. With respect to the cell mock community, we previously reported that mixtures formulated based on total DNA quantification, through measurement of adenine content in whole cells, showed better concordance with metagenome measurements, as compared to mixtures fabricated based on cell counts determined by flow cytometry (11). In addition, DNA content can also account for potential variations in genome ploidy.

After having characterized the mock communities, we next leveraged the collaborative study results to establish their fitness for purpose in two typical use scenarios. Firstly, we evaluated the utility of the mock communities to reveal differences in performance of methods and/or laboratories, covering DNA extraction, shotgun metagenomics and 16S rRNA gene amplicon sequencing. Secondly, we used the shotgun metagenomics data to evaluate different taxonomic profiling tools and to assess the impact of trimming and filtering (that is, preprocessing) of reads on downstream analysis results.

### Comparison of protocols and laboratories.

As summarized in Table S2 and Fig. S1 in the supplemental material, each of the participating laboratories analyzed the DNA and cell mock communities, performing DNA extraction, library construction and sequencing. Extraction of DNA from the cell mock community was performed either following our previously established SOP (protocol N) or using custom protocols (see Supplementary Methods for details). Libraries for shotgun metagenomics, from both the DNA mock community and extracted DNA for the cell mock community, were generated by three laboratories, following either our SOPs (protocols B and K) or using alternative methods based on commercial kits. Further, all laboratories also generated amplicon libraries of the V4 hypervariable region of the 16S rRNA gene, following either Illumina’s standard two-step tailed PCR protocol with KAPA HiFi DNA polymerase, which we here considered as SOP, or using locally preferred protocols using different polymerases (see Supplementary Methods for details, Table S2).

For the shotgun metagenome data, abundances of the strains were determined by kallisto as above. For the 16S rRNA gene amplicon data, reads were trimmed based on quality and filtered, paired reads merged, and the abundance of each strain estimated using USEARCH’s annot command, requiring a sequence identity of ≥97% to the reference sequences. For comparison with the metagenomics-based species profiles, sequence counts for the amplicon data were corrected for 16S rRNA gene copy number and converted to relative abundances.

**FIG 2:**
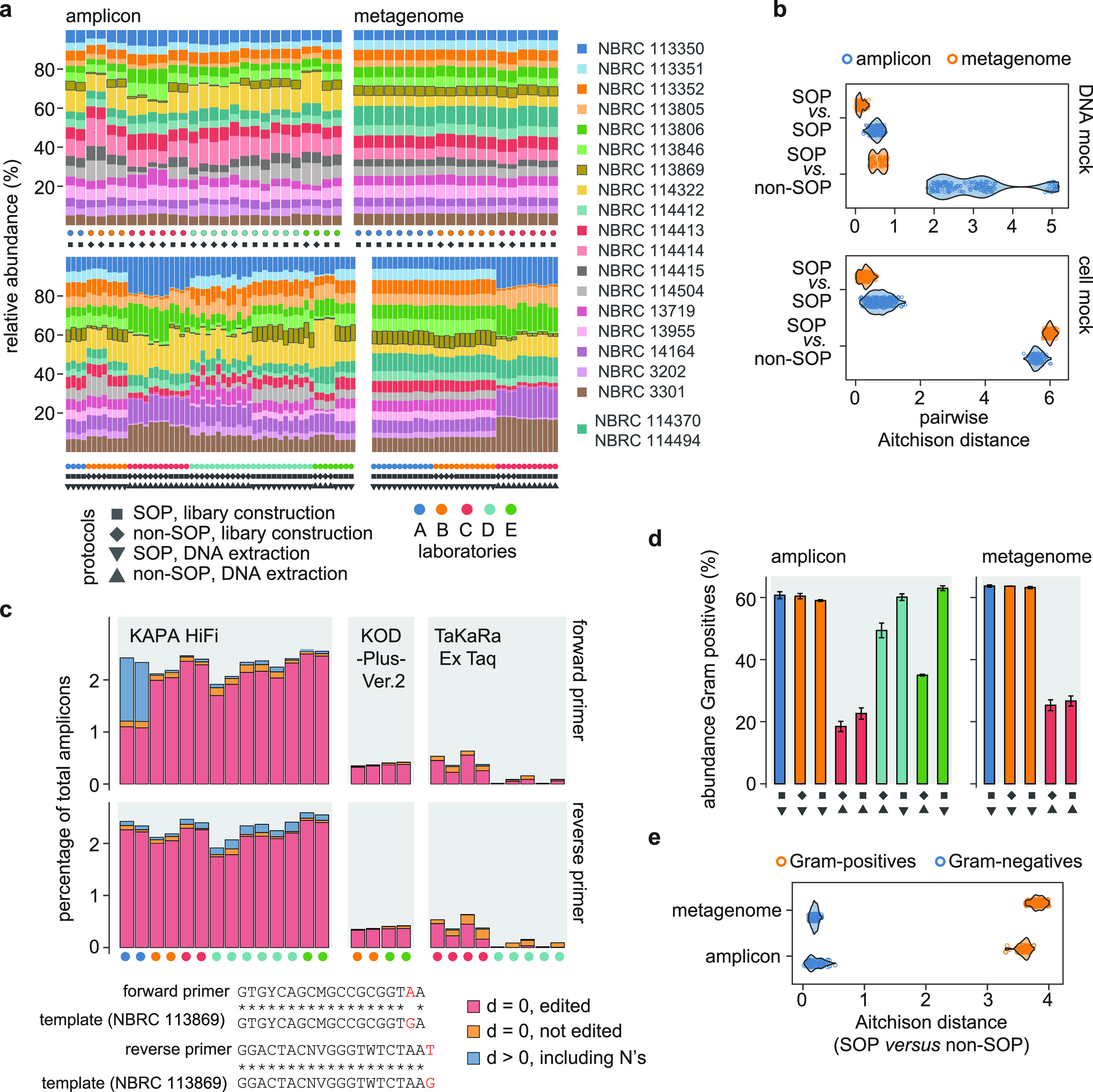
(a) Stacked bar charts of individual measurement results generated in the collaborative study for the DNA (top) and cell (bottom) mock community by amplicon and metagenome sequencing. Symbols below each bar indicate the type of protocol (SOP *versus* non-SOP for DNA extraction and library construction) and laboratory, as indicated in the legend. Note that the two strains of Bifidobacterium longum (NBRC 114370 and NBRC 114494) were analyzed as a single species. (b) Violin plots of pairwise Aitchison distances for measurements performed using SOPs and non-SOPs for library construction (top) and DNA extraction (bottom). Individual data points were overlaid with jitter. For the cell mock community, only protocols employing SOPs for library construction were included to highlight the effect of DNA extraction. (c) Bar charts of the relative abundance (without 16S rRNA gene copy number correction) of *C. acnes* strain NBRC 113869 for amplicon libraries prepared following the SOP using KAPA HiFi DNA polymerase and non-SOPs using alternative polymerases as indicated. Red and orange bars represent amplicons perfectly matching the expected primer sequences (Hamming distance, d, of zero), with and without editing, respectively. Blue bars indicate amplicons with at least a single mismatch, including undetermined (N) bases, to the expected edited or unedited primer sequences. The location of the primer mismatches to the template sequences is shown below the plot. (d) Cumulative relative abundance of Gram-positives measured for the cell mock community by amplicon and metagenome sequencing. Bars are colored by laboratory and symbols below the bars show the type of protocol, as in panel a. Bar heights represent the mean and error bars show standard deviations. (e) Violin plots of the distribution of pairwise Aitchison distances for the subcomposition of Gram-positives and negatives, considering SOPs and non-SOPs for DNA extraction and SOPs only for library construction.

The DNA mock community revealed high reproducibility of species profiles generated by different laboratories when using SOPs for both metagenome and amplicon library construction (Fig. 2a). To quantify differences between replicates, we examined variability in measured compositions based on Aitchison distances. While similar analysis using Bray-Curtis dissimilarities yielded qualitatively consistent results, the Aitchison distance is considered a superior metric because it better accounts for compositionality of the data. This analysis showed that between-laboratory dispersion of species profiles measured by amplicon sequencing was, as expected, higher than for metagenome sequencing (*P*-value < 0.01; Mann-Whitney U test of pairwise Aitchison distances; Fig. 2b). Use of non-SOPs for library construction had only a small effect on species profiles measured by metagenomics but led to much larger differences for amplicon sequencing. As assessed using the cell mock community, the SOP for DNA extraction similarly yielded highly reproducible results across laboratories, both as measured by metagenome and amplicon sequencing (Fig. 2b). On average, use of non-SOPs for DNA extraction led to the largest variability in species profiles, underscoring that DNA extraction represents a dominant source of variability in metagenomic analysis methods.

In addition to serving as control reagents for comparing results across methods or laboratories, the mock communities may also assist in identifying systematic bias due to specific protocols. For instance, we found that the abundance of strain *C. acnes* NBRC 113869 was significantly higher in amplicon libraries generated using the SOP compared to the evaluated non-SOPs (*P*-value < 0.01, Welch’s *t* test), and consistent with abundances estimated by metagenomics (Fig. 2a). Based on previous studies (12, 25), it was hypothesized that the superior recovery of *C. acnes* by the SOP was associated with the use of KAPA HiFi DNA polymerase because this enzyme can efficiently amplify sequence templates with mismatches to the primers by so-called primer editing. Indeed, inspection of the primer regions in the sequenced amplicons showed that, on average, 96% of sequences were edited to match the template sequence (Fig. 2c). While KOD-Plus- DNA polymerase also showed high primer-editing activity, overall recovery of *C. acnes* remained however much lower (Fig. 2c). Based on this finding, and in line with the best practices of Gohl et al. (12), the use of polymerases with strong proof-reading/primer-editing activity is thus recommended for amplicon-based microbiome studies, at least provided that non-target amplification, due to for example excess of host DNA in tissue-derived samples, is not of concern or can be suppressed. This is expected to improve accuracy by enabling efficient amplification of sequence templates with mismatches to the used PCR primers, especially mismatches near the 3′-end of commonly used 16S rRNA gene amplification primers (25).

In similar fashion, the cell mock community revealed that differences in species profiles between the SOP and non-SOPs for DNA extraction were mainly due to variable total recovery of Gram-positives, as consistently observed by metagenome and amplicon sequencing (Fig. 2d). Further, differences in species profiles within the subcomposition of Gram-positives were much larger than within the subcomposition of Gram-negatives (Fig. 2e). This indicated that the efficiency of DNA recovery between Gram-positive species/strains is more variable than for Gram negatives, as has been reported previously by us (11) and others (15).

Taken together, these data showed that the mock communities can reveal technically meaningful differences in measurement results due to varying protocols and laboratories. They thus provide valuable control reagents to improve consistency, especially when used in conjunction with defined acceptance criteria for validating protocols and routine quality assurance. For instance, application of the above-defined guidance values showed that all here evaluated non-SOPs for DNA extraction resulted in higher than allowable errors whereas non-SOPs for metagenome library construction showed only slightly different performance than the SOPs (Fig. S4 in the supplemental material). Further, the mock communities also revealed important sources of bias and thus provide a useful resource for development and optimization of protocols.

### Comparison of taxonomic profilers and impact of read processing.

Mock communities can further serve as control reagents for assessing performance of bioinformatics procedures. Here, we demonstrate this by comparing different taxonomic profiling tools and evaluating the effect of quality trimming and filtering of reads on species abundances.

**Comparison of taxonomic profilers.** To compare metagenome-based taxonomic profilers, we used the sequencing data generated as part of the characterization of the mock communities, providing a total of 16 data sets for the DNA mock community as detailed above (Fig. S1 and Table S3 in the supplemental material). To account for differences in sequencing depth, data were randomly subsampled to 4 million read pairs per sample, unless stated otherwise. Subsampled reads were then subjected to profiling by MetaPhlAn3 (23), mOTUs2 (26), kraken2 (27), and kraken2 followed by bracken (28). We note that this selection of tools was not intended to serve as a comprehensive benchmark but rather capture algorithms that are widely used in the microbiome field and showed overall favorable performance in recent comparisons (29, 30). For mOTUs2 and MetaPhlAn3, we used the databases provided with the tools. For kraken2 and bracken, we obtained publicly available indexes and k-mer distributions build based on the Genome Taxonomy Database (GTDB, release 95; referred to as kraken2_gtdb and bracken_gtdb hereafter) and RefSeq (referred to as kraken2_refseq, and bracken_refseq). Further, for kraken2, we applied a relatively lenient threshold (0.05) for the confidence score, following a recent benchmarking study (29). For bracken, we set the abundance threshold to 1000, such that species with less than 1,000 reads (prior to read reassignment) were excluded from the final species profiles.

**FIG 3:**
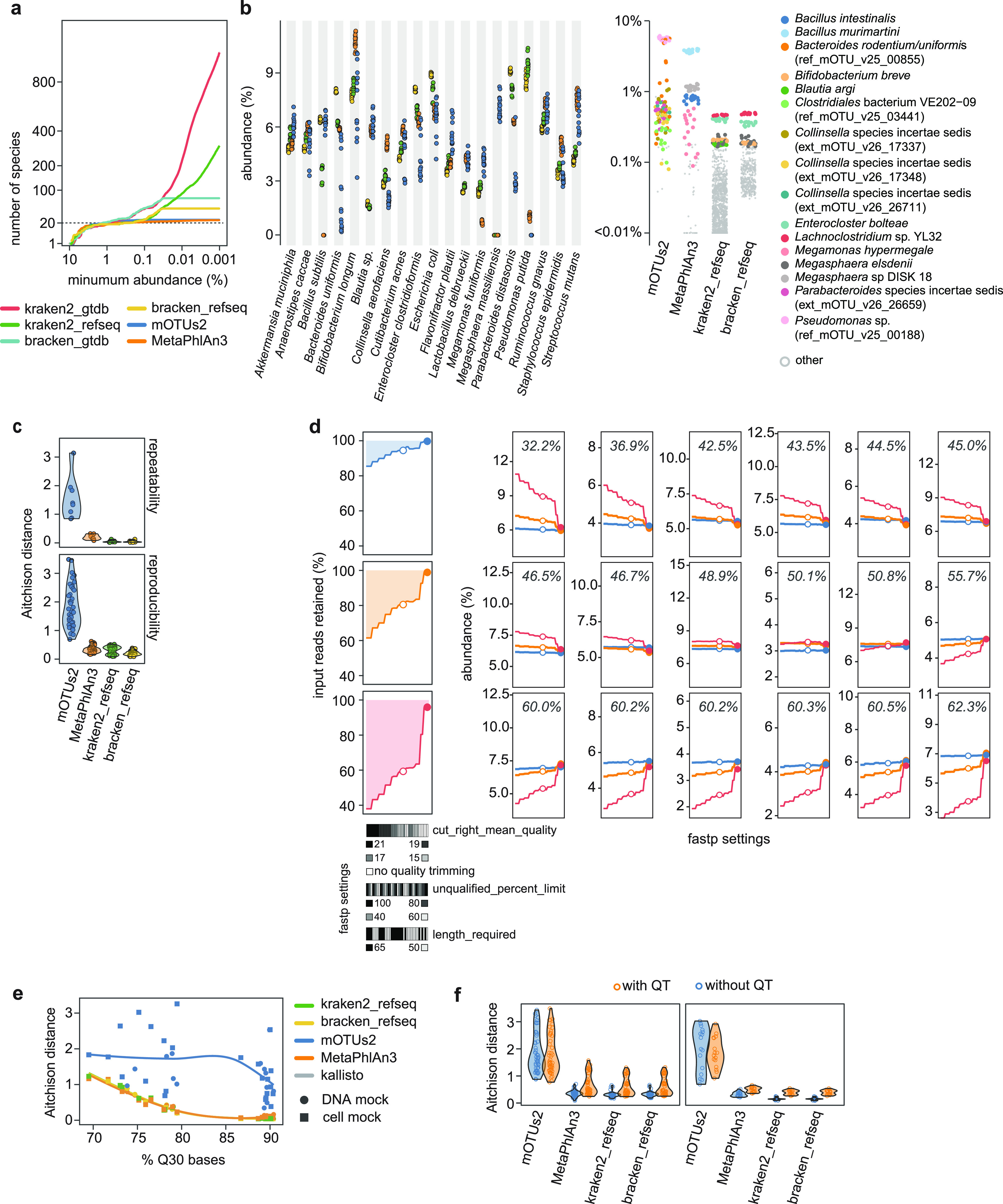
(a) Number of species classified by each of the profilers plotted as a function of minimum abundance threshold. Data are shown as the mean (colored solid lines) and standard deviation (ribbons, if visible) of 16 replicated measurement of the DNA mock community, following SOPs for library construction. The dashed horizontal line indicates the expected number of species. (b) Estimated abundances of expected species in the DNA mock community. Symbols represent results of individual measurements and are colored according to the taxonomic profiler as in panel a. The graph on the right shows the abundance of false positives (that is, species that are not included in mock the community but classified as present), with the abundant species colored as indicated in the legend. (c) Violin plots showing the distribution of Aitchison distances, calculated based on the abundances of the expected species as shown in panel b, for each of the profilers. The top and bottom plots show all pairwise dissimilarities between technical replicates within a single laboratory (denoted as repeatability) and replicates from different laboratories (denoted as reproducibility), respectively. Larger pairwise distances indicate higher variability across replicated measurements. (d) Impact of read trimming/filtering on read retention and species abundances. The left three panels show the percentage of raw reads retained following read processing by fastp for three representative data sets with varying base quality (blue: Q30 bases of 90.1%; orange: 78.2%; red: 71.7%). Applied settings for read processing by fastp, are indicated below (also see Table S5 in the supplemental material) and sorted according to the percentage of retained reads for the data set with lowest raw base quality. The adjacent plots show the relative abundance of each of the strains, as determined by kallisto, for the same three data sets. Facets are sorted according to increasing genomic GC content, indicated in each of the plots. Filled and empty circles indicate the two settings evaluated for panels d and e. (e) Aitchison distances between species profiles of read data with quality trimming (settings 15_100_65 in Table S5 in the supplemental material) and without quality trimming (settings 0_100_65). Data points, each representing a different library, are plotted as a function of the libraries’ raw base quality (*x* axis); colors and shapes reflect the mock community and profiling tool, respectively. Solid lines represent loess fits, for visualization purposes only. (f) Violin plots of pairwise Aitchison distances demonstrating the impact of quality trimming (settings 15_100_65, with QT and 0_100_65, without QT) on perceived reproducibility. Only pairwise comparisons for measurements performed by different laboratories following the SOPs were included.

To gauge precision of the profilers, we evaluated the number of species classified at varying minimum relative abundance thresholds (29). As displayed in Fig. 3a, all profilers classified a comparable number of high-abundance species, close to the expected value. Further, for the marker-gene based methods mOTUs2 and MetaPhlAn3, the number of identified species remained relatively constant for decreasing minimum abundance thresholds. In contrast, kraken2 led to a rapid proliferation of the number of identified species at lower abundances. This was especially pronounced for kraken2_gtdb, which classified a considerably number of species with relative abundances on the order of 0.1%. As the DNA mock community contains only 20 species, virtually all these species thus represent false positives (that is, species incorrectly classified as present in the mock community). Processing of the kraken2 profiles by bracken, as expected, substantially reduced the number of low-abundance species. Inspection of the species profiles further showed that false positives for kraken2, and by extension bracken, were often due to species belonging to the same genus as species known to be present in the mock community (Fig. S5 in the supplemental material).

We further compared profilers based on detection of expected species (that is, true positives as identified based on the assigned species names for each of the profilers) and agreement with the expected composition. Note that for kraken2 and bracken, we focused on profiles based on the RefSeq database (that is, kraken2_refseq and bracken_refseq) because of the challenge of handling the vast number of false positives for kraken2_gtdb. We stress however that the inflation of false positives for kraken2_gtdb is not indicative of poor performance of the GTDB *per se* but rather highlights the challenge of using comprehensive databases with read assignments by kraken2. For all profilers, expected species accounted for the majority of tallied species abundances, ranging from 92.4 ± 0.6% (mean and sd) for mOTUs2 to 97.0 ± 0.2% for bracken_refseq. As shown in Fig. 3b, estimated species abundances varied however considerably among tools and not all expected species were identified by all tools. For instance, MetaPhlAn3 failed to detect B. subtilis but rather identified two other, false positive Bacillus species, namely, B. intestinalis and B. murimartini (see also Fig. S6 in the supplemental material). Similarly, the species Megasphaera massiliensis was not identified by kraken2_refseq and bracken_refseq but rather classified as predominantly M. elsdenii (Fig. S6). As depicted in Fig. 3b, abundant ‘unexpected’ taxa typically represented species belonging to the same genus as genera present in the mock communities or taxa not fully annotated to the species level (e.g., Pseudomonas sp. for mOTUs2, *Megaspharea* sp. DISK 18 for MetaPhlAn3 and *Lachnoclostridium* sp. YL32 for kraken2_refseq). Agreement with the expected even species profiles, expressed as Bray-Curtis similarities, ranged from 78.6 ± 0.2% (bracken_refseq) to 85.0 ± 0.4% (MetaPhlAn3), which was substantially lower than for kallisto (95.6 ± 0.2%; Fig. S7 in the supplemental material).

Further, Fig. 3b also revealed higher variation in estimated species abundances across replicates for mOTUs2, with a variability of 29.2% (qmCV calculated around the grand mean of all measurements), compared to 5.2% (MetaPhlAn3) or less for the other tools. This was also reflected in terms of pairwise Aitchison distances, with mOTUs2 showing significantly larger dispersion between replicates than the other profilers (*P*-value < 0.01, Mann–Whitney U test on pairwise distances), both within a single laboratory and across laboratories (Fig. 3c). To clarify the source of this increased dispersion, we randomly subsampled the four deeply sequenced HiSeq libraries for the DNA mock community (Table S3 in the supplemental material) to between 0.5 and 20 million read pairs per sample and calculated differences between species profiles generated using the subsampled reads, for each library and subsampling depth separately. As shown in Fig. S8, mOTUs2 resulted in consistently higher differences between pairs of species profiles of subsampled reads, and this was correlated with subsampling depth. This observation suggested that the apparent poor replication for mOTUs2 seems to be due to stochastic noise arising from the smaller fraction of input reads mapped to a limited number (up to 10) of marker genes. In line with this, MetaPhlAn3, which uses about 80 clade-specific marker genes per species, also resulted in slightly higher dissimilarities than kraken2_refseq and bracken_refseq (Fig. S8) but this effect was less pronounced than for mOTUs2. These observations suggest that deeper sequencing is beneficial for profiling tools that use a limited number of marker genes to improve reproducibility of inferred species abundances.

**Species-dependent bias due to read trimming and filtering.** To assess the impact of read trimming and filtering, we exploited the variable raw base quality across NextSeq 500 sequencing runs and libraries (Table S3 in the supplemental material). More specifically, unless stated otherwise, we processed the reads with fastp using a range of settings for the parameters cut_right_mean_quality, length_required and unqualified_percent_limit (Table S5). As depicted in Fig. 3d, the applied settings resulted in highly variable retention of raw reads, with lower-quality reads being discarded more aggressively when applying more stringent read trimming and filtering.

To inspect the impact of read trimming and filtering, we plotted the relative abundance of each strain in the processed data as a function of fastp settings, from more to less stringent (Fig. 3d). This revealed substantial differences in strain-wise abundances for the poorest quality read data upon more aggressive filtering/trimming of raw reads, and this effect was strongly associated with the genomic GC content of the strains. More specifically, reads from GC-rich genomes tended to be removed more aggressively (Fig. S9 in the supplemental material), leading to lower relative abundance of high-GC strains in the surviving reads after stringent trimming and filtering (Fig. 3d). This GC-dependent effect was also observed after processing of the reads using Cutadapt (Fig. S10), which employs a different algorithm for trimming of reads based on their base quality scores than fastp.

Increased bias due to read trimming and filtering was further consistently observed in species profiles generated using the above-described taxonomic profilers. More specifically, Aitchison distances between profiles generated based on reads that underwent only minimal preprocessing (settings 0_100_65; that is, no quality filtering and minimum length of 65 bp after adapter and trailing polyX trimming) and reads subjected to quality trimming (settings 15_100_65; that is, including 4 bp window-based trimming with quality threshold of 15) increased substantially for read data with lower bases qualities (Fig. 3e). As expected, this also led to larger differences between results generated by different laboratories due to variability in base quality (Fig. 3f). Finally, although omitting read trimming led to a slight increase in the number of unclassified reads (kraken2) or “unassigned” species (mOTUs2), no strong effect on the number of classified species was found (Fig. S11 in the supplemental material).

Finally, we also observed species-dependent variation in the quality of 16S rRNA gene amplicon sequencing reads. For instance, the reverse reads of Parabacteroides distasonis NBRC 113806 consistently displayed the lowest quality (Fig. S12 in the supplemental material), measured in terms of the expected errors as predicted based on the quality scores (31). This appeared to be due to a steep decline in raw base quality around position 65 (Fig. S13), presumably due excessive dephasing during sequencing. Of note is that, within our data set, such a drop in quality was not observed for reads generated with V3 chemistry by lab E; all other labs used V2 chemistry. These data highlighted that variation in raw base quality profiles among different species can lead to appreciable bias in taxonomic profiles depending on the applied settings for processing of raw reads, as we and others documented previously (32, 33).

Taken together, the above results validated the utility of the mock communities for assessing performance of bioinformatics pipelines. The substantial deviation from the expected composition observed for all profilers also underscored that for optimal use of the mock communities, quantification should employ dedicated reference sequences, preferably complete genomes, as this can reveal more subtle and meaningful differences between protocols. Here, we employed kallisto for quantification because of its speed and accuracy but other algorithms may also be used.

Further, our observations also point to a need to sufficiently scrutinize the effect of read quality trimming and filtering on metagenomics inferences, mirroring previous recommendations for RNA-seq (34). Here, we found that, for NextSeq 500 sequencing data, species-dependent bias due to read trimming and filtering was strongly associated with genomic GC content. In-depth investigation of whether similar effects occur on other sequencing platforms was beyond the scope of this study but variable GC-related bias depending on platform has previously been reported in the context of microbial (meta)genome sequencing (35). In any case, these observations suggest that read trimming may lead to substantial bias depending on the quality of the data, which may vary among sequencing runs. As such, it is advisable to perform only gentle trimming and/or filtering of reads, provided that the performance of downstream analyses is not negatively affected. In this context, inclusion of mock communities in each sequencing run will allow consistent identification of potentially aberrant sequencing runs and adequate evaluation of settings for preprocessing of reads to prevent bias.

To conclude, we have developed two mock communities intended to serve as control reagents for human microbiota analyses by high-throughput sequencing methods. Through a collaborative study, we carefully characterized the composition of the mock communities and demonstrated their utility in typical use cases. The mock communities provide appropriate control reagents that can be analyzed in each sequencing run to track consistency of the data and reveal potential systematic bias or drift due to e.g., varying base quality and read trimming or filtering. The mock communities, which are available from the NITE Biological Resource Center (NBRC) at the National Institute of Technology and Evaluation (NITE, Japan), are expected to improve standardization and quality assurance as microbiome research is rapidly being translated into new therapeutic and diagnostic applications.

## MATERIALS AND METHODS

### Bacterial strains and cultivation.

Cells of all strains in the mock communities were obtained from NBRC at the National Institute of Technology and Evaluation (NITE, Japan). Liquid cultures were prepared using the media and cultivation conditions shown in Table S6. Cells were harvested during the late-log to stationary growth phase by centrifugation (15 min at 4,000 × *g*), washed with phosphate-buffered saline (PBS, pH 7.4), and stored at −80°C in PBS with 15% glycerol as cryoprotectant.

### Formulation of the DNA mock community.

High-molecular-weight DNA was extracted from the cultured cells using the MagAttract HMW DNA Kit (Qiagen), following the manufacturer’s instructions. To improve lysis efficiency, cells were pretreated with 8.3 mg mL^−1^ of lysozyme (Sigma) and 2800 units mL^−1^ of achromopeptidase (FUJIFILM Wako Pure Chemical Corporation) for 15–60 min at 37°C, for strains NBRC 114412, NBRC 114370, NBRC 114494, NBRC 113351, NBRC 114504, NBRC 113805, NBRC 3202, NBRC 114413, NBRC 13955, NBRC 113869, NBRC 113846, NBRC 13719, NBRC 113806, and NBRC 114322. Purified DNA was treated with 0.5 mg mL^−1^ of RNase A (Nippon Gene Co., Ltd.) in Buffer AE (Qiagen; 10 mM Tris-HCl, 0.5 mM EDTA, pH 9.0) for 30 min at 37°C and cleaned up with phenol:chloroform:isoamyl alcohol (25:24:1, vol/vol) and chloroform. Cleaned-up DNA was recovered by ethanol precipitation, dissolved in Buffer EB (Qiagen; 10 mM Tris-HCl, pH 8.5), and stored at 4°C until use.

Total DNA concentrations were measured with the Quant-iT PicoGreen dsDNA Reagent (Thermo Fisher) using a Varioskan LUX Multimode Microplate Reader (Thermo Fisher). Measurements were performed in triplicate and averaged. DNA mass concentrations were converted to genome copy numbers based on the known size (or molecular weight) of the genomes. Genomic DNA from each strain were combined to obtain a near-even DNA mock community. Aliquots at a concentration of approximately 50 ng μl^−1^ were prepared in Buffer EB and stored at −80°C until use.

### Formulation of the cell mock community.

For the cell mock community, total DNA concentrations in the cell stocks of each strain were determined by measuring adenine content following the method of de Bruin and Birnboim (22), with minor modifications as detailed previously (11). Two replicate measurements were performed and averaged. Based on the known base composition and size of the genomes, total DNA concentrations (in terms of genome copy numbers) were calculated. Cells from each strain were combined to obtain an even cell mock community, containing an equal genome copy number, or cell equivalents, of each strain. Aliquots at a concentration of approximately 4 × 10^10^ cells mL^−1^, in PBS with 15% glycerol, were prepared and stored at −80°C until use.

### Whole-genome sequencing and assembly.

Long-read libraries for Nanopore sequencing were prepared with the Ligation Sequencing Kit (SQK-LSK109, Oxford Nanopore Technologies, ONT) and Native Barcoding Expansion pack (EXP-NBD104), following manufacturer’s provided instructions. For strains NBRC 113846, NBRC 113869, NBRC 114322, NBRC 114370, NBRC 114412, NBRC 114413, NBRC 114414 and NBRC 114415, sequencing was performed on a MinION device (ONT) using an R9.4.1 flow cell. Bases were called with Guppy v4.5.3 in high accuracy mode (dna_r9.4.1_450bps_hac.cfg). For demultiplexing and trimming of barcodes, Guppy’s guppy_barcoder script was used, specifying command line flags –barcode_kits “EXP-NBD104” –trim_barcodes –num_extra_bases_trim 2. For strains NBRC 113805, NBRC 114494 and NBRC 114504, sequencing was performed on a GridION device (ONT), also using an R9.4.1 flow cell. Base calling and adapter trimming were performed using Guppy v1.8.5 and Porechop v0.2.3 (36, https://github.com/rrwick/Porechop), respectively. In all cases, ONT reads underwent additional trimming using Porechop v0.2.4, specifying option –discard_middle. Subsequently, a subset of high-quality reads with an estimated depth of 200× was selected using Filtlong v0.2.0 (https://github.com/rrwick/Filtlong), with options –min_length 1000 –min_mean_q 9 –mean_q_weight 30.

For all strains, short-read libraries were prepared using the TruSeq DNA PCR-Free kit (Illumina), following recommended procedures, and sequenced on an Illumina MiSeq instrument using V3 chemistry, generating 2 × 301 bp reads. Sequencing reads were quality controlled using fastp v0.20.0 (37), specifying options –trim_tail1 1 –trim_tail2 1 –cut_right –cut_right_window_size 4 –cut_right_mean_quality 18 –n_base_limit 0 –length_required 50.

For all strains, long-read assemblies were generated using Flye v2.8.3 (38), specifying the expected genome size (–genome-size) and flags –asm-coverage 50 –plasmids, except for strain NBRC 114322 for which option –meta was specified. For strains NBRC 114494 and NBRC 114504, final assemblies were generated using Unicycler v0.4.7 (39), using the quality-controlled Illumina short reads and Flye assemblies as inputs. For the other strains (i.e., NBRC 113846, NBRC 113869, NBRC 114322, NBRC 114370, NBRC 114412, NBRC 114413, NBRC 114414, NBRC 114415, and NBRC 113805), the long-read assemblies were polished with the long reads using Racon v1.4.20 (40), specifying options -m 8 -× -6 -g -8 -w 500. For all strains except NBRC 113805, this was followed by polishing using ONT’s Medaka v1.2.2 (https://github.com/nanoporetech/medaka). Finally, for all strains, including NBRC 113805, additional polishing was performed with the short reads using Pilon v1.23 (41); circular sequences were rotated between polishing rounds to ensure effective polishing of the ends of the sequences, including filling in a small number of bases typically missing from the ends of assemblies generated by Flye.

Genome assemblies were inspected with CheckM v1.1.3 (42), using lineage-specific marker gene sets (lineage_wf command). A phylogenetic tree was generated with the infer command of GTDB-Tk v1.6.0 (43), using the aligned single copy marker genes generated by the classify_wf command as inputs. Ribosomal RNA (rRNA) gene sequences were extracted from the genomes using Barrnap v0.9 (https://github.com/tseemann/barrnap). Recovered small subunit (16S) rRNA gene sequences were trimmed to the expected amplicon sequences (V4 hypervariable region, primer set 515F/806R, see below) using Cutadapt v3.4 (44) and unique sequence variants retained as reference sequences for analysis of the 16S rRNA gene amplicon sequencing data as detailed below.

Simulated reads were generated using BBMap’s (v38.82; 45) randomreads.sh script, specifying command line flags adderrors=f paired=t minlength = 151 maxlength = 151. For consistency with the ‘real’ read data, simulated reads were processed by fastp, with command line flags as specified above.

### Protocol (SOP) for DNA extraction.

Extraction of DNA was performed using the ISOSPIN Fecal DNA kit (Nippon Gene Co., Ltd.), following our previously developed SOP based on the manufacturer’s provided instructions (denoted as protocol N by Tourlousse et al.) (11). More specifically, sample (here, 150 μl of cell mock community), along with 700 μl of FE1 Buffer and 10 μl of RNase, were added to the provided tubes with beads. Cells were disrupted by bead-beating for 1 min at a speed of 6 m s^−1^ using a FastPrep-24 instrument (MP Biomedicals). Bead beating was repeated three times, keeping the sample at room temperature for 5 min between rounds. Subsequently, 90 μl of FE2 Buffer was added and the sample centrifuged for 15 min at 12,000 × *g*. Supernatant (up to 500 μl) was then recovered and mixed with 0.4 volumes of FB Buffer and isopropanol. The sample was finally loaded onto the spin columns and cleaned up as per the provided instructions; purified DNA was eluted in 100 μl of Tris-EDTA (pH 8.0) buffer. Detailed descriptions for the additional DNA extraction protocols (that is, non-SOPs) are provided in the Supplementary Methods.

### Protocols (SOPs) for shotgun library construction and sequencing.

Unless stated otherwise, construction of metagenome sequencing libraries was performed using the QIAseq FX DNA Library kit Qiagen; protocol B in Tourlousse et al. (11) and SMARTer ThruPLEX DNA-Seq kit (TaKaRa Bio; protocol K), according to manufacturer’s provided instructions as follows.

For protocol B, enzymatic fragmentation reactions (50 μl) contained 1× FX Buffer, 10 μl of FX Enzyme Mix, and 50 ng of DNA template; reactions were incubated for 12 min at 32°C. For adapter ligation, 5 μl of adapters were added, along with 20 μl of DNA Ligase Buffer, 10 μl of DNA Ligase, and 15 μl of RNase-free H_2_O; reactions were incubated for 15 min at 20°C. Adapter-ligated fragments were then purified and size-selected with the Agencourt AMPure XP PCR Purification system, using sequentially 1 and 0.8 volumes of bead solution, and eluted in 10 mM Tris-HCl buffer. Subsequently, adapter-ligated and size-selected fragments were amplified in PCRs (50 μl) containing 1× HiFi PCR Master Mix, 1.5 μl of Primer Mix and 23.5 μl of DNA template. Thermal cycling conditions were as follows: 98°C for 2 min; 8 cycles of 98°C for 20 s, 60°C for 30 s and 72°C for 30 s; 72°C for 1 min. Finally, amplified libraries were purified as above, using 1 volume of bead solution, and eluted in 10 mM Tris-HCl (pH 8.0).

For protocol K, 1 μg of DNA was subjected to focused ultrasonication using the Covaris M220 instrument, setting a target fragment size was 350 bp. Fragmented DNA was purified using the Agencourt AMPure XP PCR Purification system, bead-to-sample ratio of 1.8:1, and eluted in low-EDTA Tris-HCl buffer (10 mM Tris-HCl, 0.1 mM EDTA, pH 8.0). Subsequently, DNA was subjected to ‘template preparation’ in reactions (13 μl) containing 50 ng of fragmented DNA (10 μl), 2 μl of Template Preparation D Buffer and 1 μl of Template Preparation D Enzyme; reactions were incubated for 25 min at 22°C and then for 20 min at 55°C. For ‘library synthesis’, 1 μl each of Library Synthesis D Buffer and Library Synthesis D Enzyme were added and reactions incubated for 40 min at 22°C. For ‘library amplification’, 25 μl of Library Amplification D Buffer, 1 μl Library Amplification Enzyme, 5 μl of Indexing reagents and 4 μl of nuclease-free water were added and reaction subjected to thermal cycling as follows: 3 min at 72°C, 2 min at 85°C, 2 min at 98°C, 4 cycles of 98°C for 20 s, 67°C for 20 s, 72°C for 40, and 6 cycles of 98°C for 20 s and 72°C for 50 s. Finally, amplified libraries were purified with the Agencourt AMPure XP PCR Purification system, using 1 volume of beads, and eluted in 10 mM Tris-HCl (pH 8.0).

Libraries were quantified using the D5000 ScreenTape Assay system and 2200 TapeStation instrument (Agilent) and pooled at equimolar concentrations. Sequencing was performed using two different platforms, namely, a NextSeq 500 instrument using NextSeq 500/550 Mid Output Kit v2.5 (300 cycles, 2 × 151 bp reads) at Laboratory A and a HiSeq 2500 instrument using the Rapid SBS Kit V2 (200 cycles, 2 × 101 bp reads) at Laboratory C. All laboratories shared raw fastq files, after demultiplexing, with the central laboratory (that is, Lab A) for uniform processing and analysis, as described below.

### Protocol (SOP) for amplicon library construction and sequencing.

As SOP, we followed Illumina’s two-step tailed PCR protocol to generate amplicon libraries targeting the V4 hypervariable region of the 16S rRNA gene. More specifically, first round PCRs contained 1× KAPA HiFi HotStart ReadyMix, 500 nM (each) forward (5′-TCGTCGGCAGCGTCAGATGTGTATAAGAGACAGGTGYCAGCMGCCGCGGTAA-3′, synthesized by Hokkaido System Science Co., Ltd., the locus-specific 515F primer region is underlined) and reverse primer (GTCTCGTGGGCTCGGAGATGTGTATAAGAGACAGGGACTACNVGGGTWTCTAAT; the 806R primer region is underlined) and DNA template. Thermal cycling conditions were as follows: 95°C for 3 min; 25 cycles of 95°C for 30 s, 55°C for 30 s and 72°C for 30 s; and 72°C for 5 min. Amplicons were purified using the Agencourt AMPure XP PCR Purification system (bead-to-sample ratio of 1:1) and eluted in 10 mM Tris-HCl (pH 8.5). The Nextera XT Index Kit was then used to attach dual indexes and sequencing adapters, in PCRs (50 μl) containing 1× KAPA HiFi HotStart ReadyMix, 5 μl each of Index 1 and 2 primers, and 5 μl of purified first-round PCR products. Thermal cycling conditions were as follows: 95°C for 3 min; 8 cycles of 95°C for 30 s, 55°C for 30 s and 72°C for 30 s; 72°C for 5 min. Amplicons were purified as above and quantified using the D1000 ScreenTape Assay system and 2200 TapeStation instrument (Agilent). Amplicon libraries were pooled at equimolar concentrations and sequenced on a MiSeq instrument using V2 (2 × 251 bp) or V3 (2 × 301 bp) chemistry, in the presence of up to 30% of phiX control DNA. Detailed descriptions for the additional PCR protocols (that is, non-SOPs) are provided in the Supplementary Methods.

All laboratories shared demultiplexed fastq files with the central laboratory (i.e., Lab A) for processing and analysis, as described below.

### Shotgun sequencing data processing and analysis.

Whole metagenome sequencing reads were preprocessed using fastp v0.20.0, specifying the following command line arguments, unless stated otherwise: –trim_front1 5 –trim_front2 5 –trim_tail1 1 –trim_tail2 1 –unqualified_percent_limit 100 –trim_poly_x –poly_x_min_len 10 –n_base_limit 0 –low_complexity_filter –length_required 65. Following preprocessing, if applicable, fastq files were randomly subsampled to 4 million reads using seqtk v1.3 (https://github.com/lh3/seqtk), using a constant seed for the forward and reverse sequencing read data.

Quantification of mock community members in the sequencing data was performed using kallisto v0.46.1 (24), using its quantification algorithm (kallisto quant) run with default command line arguments. Estimated counts assigned to each strain were summed across replicons (that is, chromosomes and plasmids, if applicable), normalized based on their effective length to obtain strain-wise coverages, and converted to relative abundances.

Taxonomic profiling was performed using the following tools and databases: (i) kraken2 v2.1.1 (27) using the Genome Taxonomy Database, release 95 (46) or RefSeq (47); (ii) bracken v2.6.0 (28) for reassigning reads at the species level; (iii) MetaPhlAn3 v3.0.9 (23) using the mpa_v30_CHOCOPhlAn_201901 database; and (iv) mOTUs2 v2.6.1 (26) using the default database associated with this version. Sources of the databases are provided in Table S7 in the supplemental material.

For kraken2, a confidence score threshold (command line flag –confidence) of 0.05 was used, following a recent benchmarking study (29). For bracken, a threshold (command line flag -t) of 1000 was used to accommodate the relative high coverage of the simple mock community. For kraken2 and bracken, generated reports were converted to mpa format using the script kreport2mpa.py (https://github.com/jenniferlu717/KrakenTools), specifying the command line arguments –read_count –no-intermediate-ranks. For MetaPhlAn3, bowtie2 v2.4.1 (48) was used to map the reads against the reference database, specifying options –very-sensitive –no-unal; fastq files of the forward and reverse reads were concatenated prior to mapping. Subsequently, reads with a mapping quality below 5 were removed using samtools v1.10 (49) and the filtered read mappings were used for species-level profiling using MetaPhlAn3; note that no length filtering was employed as part of the MetaPhlAn3 pipeline but a minimal read length of 65 bp enforced during read preprocessing using fastp (see above). For mOTUs2, taxonomic profiles (mOTU level) were generated using the profile command, involving mapping of the reads (map_tax) to the marker gene database and generating profiles using calc_mgc and calc_motu. Command lines are provided in Table S7 in the supplemental material.

To identify expected species/strains in the outputs generated by each of the profilers, we parsed the species/mOTU-level assignments by grep using the species names, excluding subspecies names, shown in Table 1 as search terms, except for *Blautia* sp. NBRC 113351 (search term “Blautia producta”), Bacteroides uniformis NBRC 113350 (search term “Bacteroides uniformis|Bacteroides rodentium\\/uniformis”) and *Enterocloster clostridioformis* NBRC 113352 (search term “Enterocloster clostridioformis|Clostridium clostridioforme”).

### Amplicon sequencing data processing and analysis.

Trimming of primer sequences was performed using Cutadapt v3.4, specifying command line flags -g YCAGCMGCCGCGGTAA -G TACNVGGGTWTCTAAT –no-indels –discard-untrimmed –max-n 0 –error-rate 0.2 –minimum-length 225 –length 225. Subsequently, reads were truncated and filtered based on expected errors using the DADA2’s (v1.16; 50) filterAndTrim function, specifying the following parameters: truncLen = c(200,180), trimLeft = c(0, 0), maxN = c(0,0), maxEE = c(4, 6), truncQ = c(2). Trimmed reads were then merged using USEARCH’s (v11.0.667) (51) fastq_mergepairs command, with options -fastq_nostagger -fastq_pctid 80 -fastq_maxdiffs 999 -fastq_minovlen 100 -fastq_minmergelen 250 -fastq_maxmergelen 255. Finally, merged reads were annotated with USEARCH’s annot functionality, using the expected amplicon sequences extracted from the genomes (see above) as references. If applicable, counts assigned to different sequence variants for a given species were combined. Finally, read counts were corrected for 16S rRNA gene copy number, as determined based on the complete genome sequences, and species profiles expressed in terms of relative abundances, as percentages, based on the corrected read counts.

To investigate primer editing, we retained the leading 19 and 20 bp of the forward and reverse raw reads, respectively. This information was merged with the output of USEARCH annot, based on read names, and compared to the expected primer sequences. Similarly, to evaluate inspect strain-wise distributions of expected errors, USEARCH’s fastq_filter command was used to append the expected errors to the read names (option-fastq_eeout), which was then merged with the read annotations for downstream analyses.

### Downstream data analysis and visualization.

All data were imported into R (v4.0.2) (52) for processing and visualization. Parsing of data was performed mainly using the packages dplyr (v1.0.3) (53) and tidyr (v1.1.2) (54). All graphics were generated using ggplot2 (v3.3.3) (55).

Differences between pairs of species-level taxonomic profiles were calculated as Aitchison distances (i.e., Euclidean distances after centered log ratio, clr, transformation) or as Bray-Curtis dissimilarities, using the function vegdist in the R package vegan v2.5 (56). Variability between taxonomic profiles was calculated as the quadratic mean of species-wise coefficients of variation of abundances (denoted qmCV), as defined previously (11). Similarly, differences between two compositions (e.g., between measured and expected species profiles) were computed as the geometric mean of species-wise absolute fold differences (denoted gmAFD).

Guideline values for ‘allowable level of errors’ for the mock communities, here defined as the difference between values assigned by total DNA quantification during formulations and values measured by metagenomics, were calculated as described by Tourlousse et al. (11). In short, we simulated formulated mock communities (that is, quantified based on DNA content) using R stats’ rnorm function, using the values assigned by total DNA measurements as mean and a standard deviation of 5% and 10% for the DNA and cell mock community, respectively. For the metagenomic measurements, generated using SOPs (see main text), simulated compositions were generated using the function rnorm.acomp in the R package compositions (v2.0-1) (57). Here, the compositional center (that is, the closed geometric mean of strain-wise abundances) and (co)variance matrix (calculated using compositions’ var.acomp function) were used, setting the off-diagonal of the (co)variance matrix to zero. Subsequently, differences between both simulated data sets were calculated for 10^5^ random pairs and the 95% percentile set as the allowable level of errors. Based on the simulated data sets, we further defined ‘acceptance’ ranges for strain-wise abundances based on the absolute difference between both simulated data sets. More specifically, we calculated the 95% percentile of absolute differences and then calculated boundaries as the formulated abundances plus/minus the calculated 95% percentile.

### Data availability.

All sequencing data generated during the current study are available in NCBI’s Sequence Read Archive (SRA) under BioProject PRJNA747117; SRA accession numbers for individual data sets are provided in Table S3 in the supplemental material. Genome sequences have been deposited in DDBJ/EMBL/GenBank under the accession numbers shown in Table 1.
