# Supplementary material for: Characterization and Demonstration of Mock Communities as Control Reagents for Accurate Human Microbiome Community Measurements
Source: Microbiol Spectr. 2022 Mar 2;10(2):e01915-21. doi: 10.1128/spectrum.01915-21 (PMC8941912; doi:10.1128/spectrum.01915-21)
Supplement: SUPPLEMENTAL FILE 1 — Supplemental material. Download SPECTRUM01915-21_Supp_1_seq2.pdf, PDF file, 2.2 MB [file spectrum01915-21_supp_1_seq2.pdf]

Supplement for

Characterization and demonstration of mock communities as control reagents for accurate human microbiome community measurements

Dieter M. Tourlousse [1], Koji Narita [2,3], Takamasa Miura [4], Akiko Ohashi [1], Masami Matsuda [1], Yoshifumi Ohyama [4], Mamiko Shimamura [4], Masataka Furukawa [4], Ken Kasahara [2,3], Keishi Kameyama [5], Sakae Saito [6], Maki Goto [6], Ritsuko Shimizu [6], Riko Mishima [7], Jiro Nakayama [4,7], Koji Hosomi [8], Jun Kunisawa [8], Jun Terauchi [2,9], Yuji Sekiguchi [1,#], Hiroko Kawasaki [4,#]

[1] Biomedical Research Institute, National Institute of Advanced Industrial Science and Technology (AIST), Tsukuba, Ibaraki 305-8566, Japan

[2] Japan Microbiome Consortium (JMBC), Osaka, Osaka 530-0011, Japan

[3] Chitose Laboratory Corp., Kawasaki, Kanagawa 216-0041, Japan

[4] Biological Resource Center, National Institute of Technology and Evaluation (NITE), Kisarazu, Chiba 292-0818, Japan

[5] Institute of Food Sciences and Technologies, Ajinomoto Co., Inc., Kawasaki, Kanagawa 210-8681, Japan

[6] Department of Integrative Genomics, Tohoku Medical Megabank Organization, Tohoku University, 2-1 Seiryo-machi, Aoba-ku, Sendai, Miyagi 980-8573, Japan

[7] Department of Bioscience and Biotechnology, Faculty of Agriculture, Graduate School, Kyushu University, 744 Motooka, Nishi-ku, Fukuoka, Fukuoka 819-0395, Japan

[8] Laboratory of Vaccine Materials, Center for Vaccine and Adjuvant Research, and Laboratory of Gut Environmental System, National Institutes of Biomedical Innovation, Health and Nutrition (NIBIOHN), 7-6-8 Asagi Saito, Ibaraki, Osaka 567-0085, Japan

[9] Ono Pharmaceutical Co., Ltd., Osaka, Osaka 541-8564, Japan

[#] Corresponding authors: y.sekiguchi@aist.go.jp and kawasaki-hiroko@nite.go.jp

Supplementary Methods

Protocols (non-SOPs) for DNA extraction

For Laboratory C, extraction of DNA was performed using the DNeasy PowerSoil HTP 96 Kit (Qiagen), following the manufacturer's recommended protocol. Bead-beating was performed using a MM 400 Mixer Mill (Retsch GmbH) at approximately 1,600 rpm for 20 min.

For Laboratory D, extraction of DNA was performed by a phenol/bead-beating method. First, 300 mg of 0.1-mm glass beads, 300 µl of extraction buffer (167 mM Tris-HCl, pH 9.0, 67 mM EDTA and 1.7% SDS) and 500 µl of Tris-EDTA saturated phenol (Nippon Gene Co. Ltd., Tokyo, Japan) were added to the sample. Cells were then mechanically disrupted by bead-beating for 30 s at a power level of 5.0 in a FastPrep 24 instrument (MP Biomedicals, Irvine, CA, USA). After centrifugation at 15,000 × *g* for 5 min at 4ºC, the supernatant was transferred to a new 2.0-ml screw cap tube and mixed with 400 µl of phenol/chloroform/isoamyl alcohol (Nippon Gene Co. Ltd., Tokyo, Japan). The mixture was vortexed for 45 s at a power level of 4.0 using a FastPrep 24 and centrifuged at 15,000 × *g* for 5 min at 4ºC. The supernatant was then collected and DNA was precipitated by the addition 25 µl of 3 M sodium acetate (Nippon Gene Co. Ltd., Tokyo, Japan) and 300 µl of 2-propanol (Sigma-Aldrich Co. LLC., St. Louis, MO, USA). After incubation at -30°C for approximately 60 min, the mixture was centrifuged at 15,000 × *g* for 5 min at 4ºC, and the pellet washed with 500 µl of 70% ethanol, and dissolved in 200 µl of Tris-EDTA buffer.

For Laboratory E, extraction of DNA was performed by combining 300 µl of lysis buffer (No. 10, Kurabo Industries Ltd.) and 0.5 g of 0.1-mm glass beads were mixed with the sample, followed by mechanical disruption using a Cell Destroyer PS1000 (Bio Medical Science, Tokyo, Japan) at approximately 4,000 rpm for 50 s at room temperature. Supernatant was then collected after centrifugation at 13,000 × *g* for 5 min and treated with 150 µl of proteinase K buffer (No. 2, Kurabo Industries Ltd; containing 0.4 mg ml^{-1} of proteinase K). Purification of DNA was then performed with a Gene Prep Star PI-80X system (Kurabo Industries Ltd), following the manufacturer's provided instructions.

Protocols (non-SOPs) for metagenome library construction

For Laboratory B, shotgun metagenome libraries were constructed using the TruSeq DNA PCR-Free Kit (Illumina). To this end, DNA (1 µg) was fragmented by focused ultrasonication using a Covaris S220 instrument, with a target insert size of 550 bp. Fragmented DNA was then cleaned up and subjected to end-repair, adapter ligation and size selection, 3′-end adenylation and adapter ligation, following provided procedures.

For Laboratory C, shotgun metagenome libraries were prepared using an Illumina DNA Prep Kit (formerly, Nextera XT DNA Library Prep Kit), starting from 200 pg of input DNA, following manufacturer's provided instructions.

Protocols (non-SOPs) for amplicon library construction

For Laboratory B, first round PCR reactions (25 µl, triplicates) using KOD -Plus- Ver.2 (Toyobo Co., Ltd.) with 240 nM each of forward (5′-TCGTCGGCAGCGTCAGATGTGTATAAGAGACAGGTGYCAGCMGCCGCGGTAA-3′) and reverse (5′-GTCTCGTGGGCTCGGAGATGTGTATAAGAGACAGGGACTACNVGGGTWTCTAAT-3′) primer and 10 ng of template DNA.

Thermal cycling conditions were as follows: 94°C for 2 min; 18 cycles of 98°C for 10 s, 55°C for 30 s and 68°C for 30 s. Triplicate PCR reactions were then pooled and purified using SPRIselect magnetic beads (Beckman Coulter). Amplicons were eluted with Buffer EB (Qiagen) and quantified by Quant-iT PicoGreen dsDNA Reagent (Thermo Fisher). To attach dual indexes and sequencing adapters, second-round PCR reactions (50 µl) using KOD -Plus- Ver.2 with 180 nM each of forward and reverse primers and 5 ng of purified first-round PCR products. Thermal cycling conditions were as follows: 94°C for 2 min; 8 cycles of 98°C for 10 s, 55°C for 30 s and 68°C for 45 s. Amplicons were then purified using SPRIselect magnetic beads and eluted with Buffer EB. The DNA concentration was quantified by Quant-iT PicoGreen dsDNA Reagent.

For Laboratory C, two non-SOPs for amplicon library construction involving two-step tailed PCR were evaluated as follows. In one protocol, first round PCR reactions (10 µl) contained 1× Ex Taq buffer (Takara Bio), 200 µM of each dNTP (Takara Bio), 250 nM each for forward (5′-TCGTCGGCAGCGTCAGATGTGTATAAGAGACAGGTGYCAGCMGCCGCGGTAA-3′) and reverse primer (5′-GTCTCGTGGGCTCGGAGATGTGTATAAGAGACAGGGACTACNVGGGTWTCTAAT-3′), 0.25 units of TaKaRa Ex Taq DNA polymerase and 1 ng of template DNA. Thermal cycling conditions were as follows: 94°C for 3 min; 35 cycles of 94°C for 45 s, 50°C for 1 min and 72°C for 1.5 min; 72°C for 10 min. Amplicon were then purified using Agencourt AMPure XP PCR Purification system (0.8 volumes) and eluted in 100 µl of 10 mM Tris-HCl (pH 8.0). The Nextera XT Index Kit was then used to attach dual indexes and sequencing adapters, in PCR reactions (20 µl) containing 1× Ex Taq buffer (Takara Bio), 200 µM of each dNTP (Takara Bio), 2 µl each of Index 1 and 2 primers, 0.5 units of TaKaRa ExTaq DNA polymerase and 0.2 µl of template DNA. Thermal cycling conditions were as follows: 98°C for 30 s; 12 cycles of 98°C for 40 s, 65°C for 30 s and 72°C for 30 s; 72°C for 5 min. Amplicons were then separated on 1% agarose gel and purified using the MinElute Gel Extraction Kit (Qiagen). The DNA concentration was quantified by the Qubit dsDNA HS Assay Kit (Thermo Fisher Scientific). For the other protocol, two-step tailed PCR was performed using TruSeq indexed sequencing primers as follows: forward (5′-ACACTCTTTCCCTACACGACGCTCTTCCGATCTNNNNNNGTGCCAGCMGCCGCGGTAA-3′) and reverse (5′-GTGACTGGAGTTCAGACGTGTGCTCTTCCGATCTNNNNNGGACTACHVGGGTWTCTAAT-3′) primers in the first round, and forward (5′-AATGATACGGCGACCACCGAGATCTACAC[i5]ACACTCTTTCCCTACACGACGCTCTTCCGATCT-3′) and reverse (5′-CAAGCAGAAGACGGCATACGAGAT[i7]GTGACTGGAGTTCAGACGTGTGCTCTTCCGATCT-3′) primers in the second round of PCR.

For Laboratory D, first round PCR reactions (25 µl) contained 0.625 units of Takara Ex Taq HS polymerase (Takara Bio, Shiga, Japan), 1 × Ex Taq Buffer, 0.2 mM each of dNTP, 200 nM each of forward (5′-TCGTCGGCAGCGTCAGATGTGTATAAGAGACAGGTGYCAGCMGCCGCGGTAA-3′) and reverse primer (5′-GTCTCGTGGGCTCGGAGATGTGTATAAGAGACAGGGACTACNVGGGTWTCTAAT-3′) and 4.0 ng template DNA. Thermal cycling conditions were as follows: 95°C for 3 min; 25 cycles of 95°C for 30 s, 55°C for 30 s and 72°C for 30 s; 72°C for 5 min. The amplified products were then used as templates for second round PCR to attach dual indexes and sequencing adapters, in PCR reactions (50 µl) containing 0.25 units of Takara Ex Taq HS polymerase (Takara Bio, Shiga, Japan), 1 × Ex Taq Buffer, 0.2 mM each of dNTP, 5.0 µl each of forward and reverse indexed primers of Nextera XT Index kit (Illumina, San Diego, CA, USA), and 1.0 µl of first-round PCR products. Thermal cycling conditions were as follows: 95°C for 3 min; 10 cycles of 95°C for 30 s, 55°C for 30 s and 72°C for 30 s; 72°C for 5 min. Amplicons were purified using the Agencourt AMPure XP PCR Purification

system and eluted in 10 mM Tris-Cl (pH8.5), and quantified using a Quant-iT PicoGreen dsDNA Assay kit (Life Technologies, Carlsbad, CA, USA).

For Laboratory E, first round PCR reactions (25 µl) contained 1× PCR buffer, 0.2 mM of each dNTP, 1 mM MgSO4, 200 nM each of forward (5′-TCGTCGGCAGCGTCAGATGTGTATAAGCGACAGCCTACGGGNGGCWGCAG-3′) and reverse (5′-GTCTCGTGGGCTCGGAGATGTGTATAAGAGACAGGACTACHVGGGTATCTAATCC-3′) primer, 1 unit of KOD-Plus-v2 DNA polymerase (Toyobo Co., Ltd.) and 5 µl of template DNA. Thermal cycling conditions were as follows: 95ºC for 3 min; 25 cycles of 95ºC for 30 s, 55ºC for 30 s and 68ºC for 1 min; and 68ºC for 5 min. Amplicon were then purified using AMPure XP (Beckman Coulter, Inc.) and eluted in 50 µl of 10 mM Tris-HCl, pH 8.5. The Nextera XT Index Kit Set A was then used to attach dual indexes and sequencing adapters, in PCR reactions (50 µl) containing 1× PCR buffer, 0.2 mM of each dNTP, 1 mM MgSO4, 5 µl each of forward and reverse indexed primers, 1 unit of KOD-Plus-v2 DNA polymerase (Toyobo Co., Ltd.) and 5 µl of template DNA. Thermal cycling conditions were as follows: 95ºC for 3 min; 8 cycles of 95ºC for 30 s, 55ºC for 30 s and 68ºC for 1 min; and 68ºC for 5 min. Amplicons were then purified using the AMPure XP (Beckman Coulter, Inc.) and quantified with QuantiFluor ONE dsDNA System (Promega Co.).

Supplementary Tables

Table S1. Summary of genome sequences for the mock community members.

species, strain	assembly pipeline [a]	chromosome and plasmids	genome size (bp)	GC content (%)	coding density (%) [b]	16S rRNA genes [c]	completeness [d]	contamination [d]
S. epidermidis NBRC 113846	Flye, Racon, Medaka, Pilon	4	2,520,735	32.15	82.50	6	99.81	0.00
B. longum NBRC 114370	Flye, Racon, Medaka, Pilon	2	2,594,022	60.14	86.60	5	100.00	0.00
A. muciniphila NBRC 114322	Flye, Racon, Medaka, Pilon	2 [e]	2,788,377	55.65	88.24	3	97.96	0.00
R. gnavus NBRC 114413	Flye, Racon, Medaka, Pilon	2	3,757,469	42.49	89.40	5	99.42	0.39
A. caccae NBRC 114412	Flye, Racon, Medaka, Pilon	1	3,284,789	44.46	90.15	4	99.33	4.03
C. acnes subsp. *acnes* NBRC 113869	Flye, Racon, Medaka, Pilon	1	2,560,907	60.02	89.51	3	100.00	0.00
M. funiformis NBRC 114415	Flye, Racon, Medaka, Pilon	1	2,464,533	31.45	88.20	6	100.00	1.27
M. massiliensis NBRC 114414	Flye, Racon, Medaka, Pilon	1	2,610,024	50.59	87.68	7	100.00	0.60
F. plautii NBRC 113805	Flye, Racon, Pilon	1	4,277,038	60.35	87.90	3	99.33	0.81
B. longum subsp. *longum* NBRC 114494	Flye, Unicycler	3	2,534,372	60.11	87.08	4	100.00	0.00
C. aerofaciens NBRC 114504	Flye, Unicycler	3	2,278,612	60.28	85.81	5	100.00	0.81
Blautia sp. NBRC 113351	Unicycler	1	6,247,046	46.65	89.24	5	99.36	0.00
B. uniformis NBRC 113350		5	4,989,532	46.24	90.21	4	99.26	0.87
B. subtilis NBRC 13719		2	4,295,305	43.34	87.85	10	99.81	0.35
E. clostridioformis NBRC 113352		2	5,687,315	48.92	86.46	5	99.32	0.00
E. coli NBRC 3301		2	4,755,096	50.75	87.38	7	99.97	0.04
L. delbrueckii subsp. *delbrueckii* NBRC 3202		1	1,910,306	50.07	85.32	8	99.03	0.00
P. distasonis NBRC 113806		1	5,179,960	45.01	90.15	7	99.42	0.71
P. putida NBRC 14164		1	6,156,701	62.33	88.85	7	99.88	1.12
S. mutans NBRC 13955		1	2,018,796	36.87	85.50	5	100.00	0.00

[a] For strains (n=12) for which the assembly pipeline is shown, genome sequences were generated in this study.
[b] Coding density as estimated by CheckM (v1.1.3; https://github.com/Ecogenomics/CheckM).
[c] Small subunit (16S) rRNA genes were identified using barrnap (v0.9; https://github.com/tseemann/barrnap).
[d] Completeness and contamination were estimated using CheckM v1.1.3, using the lineage specific workflow (lineage_wf).
[e] Two linear contigs, namely a chromosome and presumed bacteriophage genome.

Table S2. Overview of protocols for DNA extraction, library construction and sequencing.

laboratory [a]	protocol(s) for DNA extraction	protocol(s) for metagenome library construction	protocol(s) for amplicon library construction	sequencing instrument (read length)
Lab A	• protocol N (SOP)	• protocol B (SOP)<br>• protocol K (SOP)	• Illumina's two-step PCR protocol with dual indexing (SOP) (DNA polymerase: Kapa HiFi)	• NextSeq (2×151 bp)<br>• Miseq (2×251 bp)
Lab B	• protocol N (SOP)	• protocol B (SOP)<br>• protocol K (SOP)<br>• TruSeq DNA PCR-Free Kit (non-SOP)	• Illumina's two-step PCR protocol with dual indexing (SOP) (DNA polymerase: Kapa HiFi)<br>• Two-step PCR protocol with dual indexing (DNA polymerase: KOD -Plus- Ver.2)	• NextSeq (2×151 bp) [b]<br>• Miseq (2×251 bp)
Lab C	• PowerSoil DNA Isolation Kit (non-SOP)	• protocol B (SOP)<br>• protocol K (SOP)<br>• Illumina DNA Prep Kit (non-SOP)	• Illumina's two-step PCR protocol with dual indexing (SOP) (DNA polymerase: Kapa HiFi)<br>• Two-step PCR protocol with dual indexing, TruSeq indexes (DNA polymerase: TaKaRa Ex Taq)<br>• Two-step PCR protocol with dual indexing, Nextera indexes (DNA polymerase: TaKaRa Ex Taq)	• HiSeq (2×101 bp)<br>• Miseq (2×251 bp)
Lab D	• protocol N (SOP)<br>• phenol / bead-beating protocol (non-SOP)	not applicable	• Illumina's two-step PCR protocol with dual indexing (SOP) (DNA polymerase: Kapa HiFi)<br>• Two-step PCR protocol with dual indexing (DNA polymerase: TaKaRa Ex Taq HS)	• Miseq (2×251 bp)
Lab E	• protocol N (SOP)<br>• bead-beating protocol (non-SOP)	not applicable	• Illumina's two-step PCR protocol with dual indexing (SOP) (DNA polymerase: Kapa HiFi)<br>• Two-step PCR protocol with dual indexing (DNA polymerase: KOD -Plus- Ver.2)	• Miseq (2×301 bp)

[a] Laboratory A represents the central laboratory that developed the SOPs, for DNA extraction and shotgun metagenomics library construction, used in this study (Tourlousse *et al.*, 2021).

[b] Sequencing of the metagenome libraries was performed by Lab A.

Table S3. Overview metagenomics sequencing libraries, DNA mock community.

library id	SRA accession	sequencing instrument	read length	raw reads	Q30 bases (%)	lab id	aliquot id	protocol id, library construction
metagenome_mockDna_labA_lib024	SRR17380242	NextSeq 500	2x150 bp	12,527,546	89.3	labA	1	SOP, protocol B
metagenome_mockDna_labA_lib025	SRR17380241	NextSeq 500	2x150 bp	11,530,784	89.9	labA	1	SOP, protocol B
metagenome_mockDna_labA_lib021	SRR17380245	NextSeq 500	2x150 bp	12,435,330	89.4	labA	2	SOP, protocol B
metagenome_mockDna_labA_lib023	SRR17380243	NextSeq 500	2x150 bp	11,893,366	89.7	labA	2	SOP, protocol B
metagenome_mockDna_labA_lib020	SRR17380246	NextSeq 500	2x150 bp	11,878,278	88.9	labA	3	SOP, protocol B
metagenome_mockDna_labA_lib022	SRR17380244	NextSeq 500	2x150 bp	13,848,464	89.5	labA	3	SOP, protocol B
metagenome_mockDna_labA_lib036	SRR17380229	NextSeq 500	2x150 bp	13,393,620	89.6	labA	1	SOP, protocol K
metagenome_mockDna_labA_lib026	SRR17380240	NextSeq 500	2x150 bp	15,046,826	90.4	labA	1	SOP, protocol K
metagenome_mockDna_labB_lib049	SRR17380214	NextSeq 500	2x150 bp	18,185,300	74.7	labB	N/A	non-SOP
metagenome_mockDna_labB_lib041	SRR17380223	NextSeq 500	2x150 bp	17,520,842	73.8	labB	N/A	non-SOP
metagenome_mockDna_labB_lib053	SRR17380210	NextSeq 500	2x150 bp	19,079,964	79.1	labB	N/A	SOP, protocol B
metagenome_mockDna_labB_lib045	SRR17380219	NextSeq 500	2x150 bp	16,012,084	79.5	labB	N/A	SOP, protocol B
metagenome_mockDna_labB_lib055	SRR17380208	NextSeq 500	2x150 bp	18,749,560	78.3	labB	N/A	SOP, protocol K
metagenome_mockDna_labB_lib047	SRR17380216	NextSeq 500	2x150 bp	18,431,408	78.9	labB	N/A	SOP, protocol K
metagenome_mockDna_labC_lib001	SRR17380129	HiSeq 2500	2x100 bp	42,049,280	95.9	labC	N/A	non-SOP
metagenome_mockDna_labC_lib004	SRR17380126	HiSeq 2500	2x100 bp	43,002,858	96.0	labC	N/A	non-SOP
metagenome_mockDna_labC_lib002	SRR17380128	HiSeq 2500	2x100 bp	48,301,032	96.6	labC	N/A	SOP, protocol B
metagenome_mockDna_labC_lib005	SRR17380125	HiSeq 2500	2x100 bp	42,199,016	96.5	labC	N/A	SOP, protocol B
metagenome_mockDna_labC_lib003	SRR17380127	HiSeq 2500	2x100 bp	41,143,552	96.5	labC	N/A	SOP, protocol K
metagenome_mockDna_labC_lib006	SRR17380124	HiSeq 2500	2x100 bp	47,595,948	96.4	labC	N/A	SOP, protocol K

Table S3, cont'd. Overview metagenomics sequencing libraries, cell mock community.

library id	SRA accession	sequencing instrument	read lenght	raw reads	Q30 bases (%)	lab id	aliquot id	protocol id, DNA extraction	protocol id, library construction
metagenome_mockCell_labA_lib032	SRR17380233	NextSeq 500	2x150 bp	12,780,820	89.5	A	1	SOP, protocol N	SOP, protocol B
metagenome_mockCell_labA_lib034	SRR17380231	NextSeq 500	2x150 bp	12,223,354	89.7	A	1	SOP, protocol N	SOP, protocol B
metagenome_mockCell_labA_lib031	SRR17380234	NextSeq 500	2x150 bp	12,966,356	89.7	A	1	SOP, protocol N	SOP, protocol B
metagenome_mockCell_labA_lib033	SRR17380232	NextSeq 500	2x150 bp	11,822,934	89.5	A	1	SOP, protocol N	SOP, protocol B
metagenome_mockCell_labA_lib019	SRR17380247	NextSeq 500	2x150 bp	13,482,092	88.7	A	1	SOP, protocol N	SOP, protocol K
metagenome_mockCell_labA_lib038	SRR17380226	NextSeq 500	2x150 bp	14,103,774	90.1	A	1	SOP, protocol N	SOP, protocol K
metagenome_mockCell_labA_lib035	SRR17380230	NextSeq 500	2x150 bp	13,129,384	90.3	A	1	SOP, protocol N	SOP, protocol K
metagenome_mockCell_labA_lib037	SRR17380227	NextSeq 500	2x150 bp	13,417,378	90.1	A	1	SOP, protocol N	SOP, protocol K
metagenome_mockCell_labA_lib028	SRR17380237	NextSeq 500	2x150 bp	12,891,230	90.0	A	2	SOP, protocol N	SOP, protocol B
metagenome_mockCell_labA_lib029	SRR17380236	NextSeq 500	2x150 bp	11,756,456	86.7	A	2	SOP, protocol N	SOP, protocol B
metagenome_mockCell_labA_lib027	SRR17380238	NextSeq 500	2x150 bp	12,597,526	90.1	A	3	SOP, protocol N	SOP, protocol B
metagenome_mockCell_labA_lib030	SRR17380235	NextSeq 500	2x150 bp	14,016,936	89.1	A	3	SOP, protocol N	SOP, protocol B
metagenome_mockCell_labB_lib050	SRR17380213	NextSeq 500	2x150 bp	15,643,996	75.2	B	N/A	SOP, protocol N	non-SOP
metagenome_mockCell_labB_lib042	SRR17380222	NextSeq 500	2x150 bp	16,927,002	73.3	B	N/A	SOP, protocol N	non-SOP
metagenome_mockCell_labB_lib048	SRR17380215	NextSeq 500	2x150 bp	16,955,864	75.9	B	N/A	SOP, protocol N	non-SOP
metagenome_mockCell_labB_lib040	SRR17380224	NextSeq 500	2x150 bp	20,903,712	69.5	B	N/A	SOP, protocol N	non-SOP
metagenome_mockCell_labB_lib054	SRR17380209	NextSeq 500	2x150 bp	14,072,002	73.0	B	N/A	SOP, protocol N	SOP, protocol B
metagenome_mockCell_labB_lib046	SRR17380218	NextSeq 500	2x150 bp	14,913,690	76.5	B	N/A	SOP, protocol N	SOP, protocol B
metagenome_mockCell_labB_lib052	SRR17380211	NextSeq 500	2x150 bp	14,784,320	79.0	B	N/A	SOP, protocol N	SOP, protocol B
metagenome_mockCell_labB_lib044	SRR17380220	NextSeq 500	2x150 bp	17,998,236	79.5	B	N/A	SOP, protocol N	SOP, protocol B
metagenome_mockCell_labB_lib043	SRR17380221	NextSeq 500	2x150 bp	15,066,684	78.2	B	N/A	SOP, protocol N	SOP, protocol K
metagenome_mockCell_labB_lib056	SRR17380207	NextSeq 500	2x150 bp	16,594,858	78.1	B	N/A	SOP, protocol N	SOP, protocol K
metagenome_mockCell_labB_lib051	SRR17380212	NextSeq 500	2x150 bp	17,340,834	78.0	B	N/A	SOP, protocol N	SOP, protocol K
metagenome_mockCell_labB_lib039	SRR17380225	NextSeq 500	2x150 bp	22,049,126	71.7	B	N/A	SOP, protocol N	SOP, protocol K
metagenome_mockCell_labC_lib007	SRR17380122	HiSeq 2500	2x100 bp	43,683,114	95.7	C	N/A	non-SOP	non-SOP
metagenome_mockCell_labC_lib010	SRR17380119	HiSeq 2500	2x100 bp	48,275,088	96.1	C	N/A	non-SOP	non-SOP
metagenome_mockCell_labC_lib013	SRR17380116	HiSeq 2500	2x100 bp	47,343,492	96.2	C	N/A	non-SOP	non-SOP
metagenome_mockCell_labC_lib016	SRR17380113	HiSeq 2500	2x100 bp	50,429,198	96.1	C	N/A	non-SOP	non-SOP
metagenome_mockCell_labC_lib008	SRR17380121	HiSeq 2500	2x100 bp	47,934,246	96.6	C	N/A	non-SOP	SOP, protocol B
metagenome_mockCell_labC_lib011	SRR17380118	HiSeq 2500	2x100 bp	40,510,808	96.5	C	N/A	non-SOP	SOP, protocol B
metagenome_mockCell_labC_lib014	SRR17380115	HiSeq 2500	2x100 bp	40,393,998	96.4	C	N/A	non-SOP	SOP, protocol B
metagenome_mockCell_labC_lib017	SRR17380249	HiSeq 2500	2x100 bp	46,006,248	96.6	C	N/A	non-SOP	SOP, protocol B
metagenome_mockCell_labC_lib009	SRR17380120	HiSeq 2500	2x100 bp	42,880,022	96.2	C	N/A	non-SOP	SOP, protocol K
metagenome_mockCell_labC_lib012	SRR17380117	HiSeq 2500	2x100 bp	44,986,704	96.2	C	N/A	non-SOP	SOP, protocol K
metagenome_mockCell_labC_lib015	SRR17380114	HiSeq 2500	2x100 bp	44,773,856	96.2	C	N/A	non-SOP	SOP, protocol K
metagenome_mockCell_labC_lib018	SRR17380248	HiSeq 2500	2x100 bp	50,153,010	95.7	C	N/A	non-SOP	SOP, protocol K

Table S3, cont'd. Overview amplicon sequencing libraries, DNA mock community.

library id	SRA accession	raw reads	Q30 bases (%)	lab id	operator id	protocol id, library construction
amplicon_mockDna_labA_lib001	SRR17380252	317,024	87.5	A	N/A	SOP
amplicon_mockDna_labA_lib002	SRR17380251	346,174	87.4	A	N/A	SOP
amplicon_mockDna_labB_lib055	SRR17380162	157,118	92.1	B	N/A	SOP
amplicon_mockDna_labB_lib056	SRR17380161	159,358	90.2	B	N/A	non-SOP, protocol ampB1
amplicon_mockDna_labB_lib057	SRR17380160	164,548	92.3	B	N/A	SOP
amplicon_mockDna_labB_lib058	SRR17380159	161,672	90.0	B	N/A	non-SOP, protocol ampB1
amplicon_mockDna_labC_lib067	SRR17380149	38,084	96.3	C	N/A	SOP
amplicon_mockDna_labC_lib068	SRR17380148	25,452	96.2	C	N/A	non-SOP, protocol ampC1
amplicon_mockDna_labC_lib069	SRR17380147	39,096	95.4	C	N/A	non-SOP, protocol ampC2
amplicon_mockDna_labC_lib070	SRR17380146	37,912	96.4	C	N/A	SOP
amplicon_mockDna_labC_lib071	SRR17380144	29,690	96.1	C	N/A	non-SOP, protocol ampC1
amplicon_mockDna_labC_lib072	SRR17380143	37,996	95.4	C	N/A	non-SOP, protocol ampC2
amplicon_mockDna_labD_lib007	SRR17380156	21,664	89.4	D	A	SOP
amplicon_mockDna_labD_lib008	SRR17380145	16,980	91.6	D	A	non-SOP, protocol ampD1
amplicon_mockDna_labD_lib009	SRR17380134	22,184	90.5	D	B	SOP
amplicon_mockDna_labD_lib010	SRR17380123	21,108	89.6	D	B	non-SOP, protocol ampD1
amplicon_mockDna_labD_lib011	SRR17380250	26,418	89.0	D	C	SOP
amplicon_mockDna_labD_lib013	SRR17380228	15,782	90.2	D	A	SOP
amplicon_mockDna_labD_lib014	SRR17380217	14,476	91.0	D	A	non-SOP, protocol ampD1
amplicon_mockDna_labD_lib015	SRR17380206	17,526	89.2	D	B	SOP
amplicon_mockDna_labD_lib016	SRR17380205	16,442	89.9	D	B	non-SOP, protocol ampD1
amplicon_mockDna_labD_lib017	SRR17380204	16,390	90.0	D	C	SOP
amplicon_mockDna_labD_lib018	SRR17380203	42,896	89.6	D	C	non-SOP, protocol ampD1
amplicon_mockDna_labE_lib043	SRR17380175	1,349,346	91.8	E	N/A	SOP
amplicon_mockDna_labE_lib044	SRR17380174	1,000,362	92.0	E	N/A	non-SOP, protocol ampE1
amplicon_mockDna_labE_lib045	SRR17380173	1,549,562	92.0	E	N/A	SOP
amplicon_mockDna_labE_lib046	SRR17380172	1,022,300	91.7	E	N/A	non-SOP, protocol ampE1

Table S3, cont'd. Overview amplicon sequencing libraries, cell mock community.

library id	SRA accession	raw reads	Q30 bases (%)	lab id	operator id	protocol id, DNA extraction	protocol id, library construction
amplicon_mockCell_labA_lib003	SRR17380200	312,540	88.1	A	N/A	SOP	SOP
amplicon_mockCell_labA_lib005	SRR17380178	348,146	87.1	A	N/A	SOP	SOP
amplicon_mockCell_labA_lib004	SRR17380189	276,526	87.5	A	N/A	SOP	SOP
amplicon_mockCell_labA_lib006	SRR17380167	362,852	86.9	A	N/A	SOP	SOP
amplicon_mockCell_labB_lib060	SRR17380157	134,720	89.7	B	N/A	SOP	non-SOP, protocol ampB1
amplicon_mockCell_labB_lib059	SRR17380158	111,836	92.3	B	N/A	SOP	SOP
amplicon_mockCell_labB_lib064	SRR17380152	171,808	92.2	B	N/A	SOP	non-SOP, protocol ampB1
amplicon_mockCell_labB_lib063	SRR17380153	135,140	92.9	B	N/A	SOP	SOP
amplicon_mockCell_labB_lib062	SRR17380154	191,600	92.3	B	N/A	SOP	non-SOP, protocol ampB1
amplicon_mockCell_labB_lib061	SRR17380155	122,778	92.7	B	N/A	SOP	SOP
amplicon_mockCell_labB_lib066	SRR17380150	200,652	91.4	B	N/A	SOP	non-SOP, protocol ampB1
amplicon_mockCell_labB_lib065	SRR17380151	105,158	92.5	B	N/A	SOP	SOP
amplicon_mockCell_labC_lib074	SRR17380141	30,084	94.8	C	N/A	non-SOP	non-SOP, protocol ampC1
amplicon_mockCell_labC_lib075	SRR17380140	36,544	94	C	N/A	non-SOP	non-SOP, protocol ampC2
amplicon_mockCell_labC_lib073	SRR17380142	37,998	95.3	C	N/A	non-SOP	SOP
amplicon_mockCell_labC_lib080	SRR17380135	27,562	94.7	C	N/A	non-SOP	non-SOP, protocol ampC1
amplicon_mockCell_labC_lib081	SRR17380133	41,302	94	C	N/A	non-SOP	non-SOP, protocol ampC2
amplicon_mockCell_labC_lib079	SRR17380136	25,742	95.4	C	N/A	non-SOP	SOP
amplicon_mockCell_labC_lib077	SRR17380138	29,380	94.5	C	N/A	non-SOP	non-SOP, protocol ampC1
amplicon_mockCell_labC_lib078	SRR17380137	39,086	94.1	C	N/A	non-SOP	non-SOP, protocol ampC2
amplicon_mockCell_labC_lib076	SRR17380139	38,494	95.4	C	N/A	non-SOP	SOP
amplicon_mockCell_labC_lib083	SRR17380131	29,092	94.8	C	N/A	non-SOP	non-SOP, protocol ampC1
amplicon_mockCell_labC_lib084	SRR17380130	36,748	94	C	N/A	non-SOP	non-SOP, protocol ampC2
amplicon_mockCell_labC_lib082	SRR17380132	26,984	95.5	C	N/A	non-SOP	SOP
amplicon_mockCell_labD_lib024	SRR17380196	23,506	91	D	C	non-SOP	non-SOP, protocol ampD1
amplicon_mockCell_labD_lib022	SRR17380198	15,934	90.2	D	B	non-SOP	non-SOP, protocol ampD1
amplicon_mockCell_labD_lib020	SRR17380201	19,928	91.2	D	A	non-SOP	non-SOP, protocol ampD1
amplicon_mockCell_labD_lib023	SRR17380197	13,752	90.1	D	C	SOP	SOP
amplicon_mockCell_labD_lib021	SRR17380199	23,418	90.8	D	B	SOP	SOP
amplicon_mockCell_labD_lib019	SRR17380202	15,894	90.4	D	A	SOP	SOP
amplicon_mockCell_labD_lib036	SRR17380183	32,200	90.7	D	C	non-SOP	non-SOP, protocol ampD1
amplicon_mockCell_labD_lib034	SRR17380185	17,852	91.5	D	B	non-SOP	non-SOP, protocol ampD1
amplicon_mockCell_labD_lib032	SRR17380187	17,518	91.2	D	A	non-SOP	non-SOP, protocol ampD1
amplicon_mockCell_labD_lib035	SRR17380184	12,330	90.9	D	C	SOP	SOP
amplicon_mockCell_labD_lib033	SRR17380186	22,070	90.2	D	B	SOP	SOP
amplicon_mockCell_labD_lib031	SRR17380188	11,934	91.3	D	A	SOP	SOP
amplicon_mockCell_labD_lib030	SRR17380190	14,228	91.4	D	C	non-SOP	non-SOP, protocol ampD1
amplicon_mockCell_labD_lib028	SRR17380192	17,870	91.4	D	B	non-SOP	non-SOP, protocol ampD1
amplicon_mockCell_labD_lib026	SRR17380194	19,634	91.7	D	A	non-SOP	non-SOP, protocol ampD1
amplicon_mockCell_labD_lib029	SRR17380191	23,860	90.7	D	C	SOP	SOP
amplicon_mockCell_labD_lib027	SRR17380193	19,492	91.1	D	B	SOP	SOP
amplicon_mockCell_labD_lib025	SRR17380195	18,416	91.1	D	A	SOP	SOP
amplicon_mockCell_labD_lib042	SRR17380176	24,742	91	D	C	non-SOP	non-SOP, protocol ampD1
amplicon_mockCell_labD_lib040	SRR17380179	17,056	90	D	B	non-SOP	non-SOP, protocol ampD1
amplicon_mockCell_labD_lib038	SRR17380181	17,480	91.6	D	A	non-SOP	non-SOP, protocol ampD1
amplicon_mockCell_labD_lib041	SRR17380177	72,482	89.8	D	C	SOP	SOP
amplicon_mockCell_labD_lib039	SRR17380180	24,592	90.5	D	B	SOP	SOP
amplicon_mockCell_labD_lib037	SRR17380182	29,078	89.6	D	A	SOP	SOP
amplicon_mockCell_labE_lib048	SRR17380170	1,033,706	92.2	E	N/A	non-SOP	non-SOP, protocol ampE1
amplicon_mockCell_labE_lib047	SRR17380171	1,404,302	92.4	E	N/A	SOP	SOP
amplicon_mockCell_labE_lib052	SRR17380165	954,568	92.7	E	N/A	non-SOP	non-SOP, protocol ampE1
amplicon_mockCell_labE_lib051	SRR17380166	1,285,102	92.1	E	N/A	SOP	SOP
amplicon_mockCell_labE_lib050	SRR17380168	1,092,300	92.4	E	N/A	non-SOP	non-SOP, protocol ampE1
amplicon_mockCell_labE_lib049	SRR17380169	1,144,812	90.5	E	N/A	SOP	SOP
amplicon_mockCell_labE_lib054	SRR17380163	945,996	92.3	E	N/A	non-SOP	non-SOP, protocol ampE1
amplicon_mockCell_labE_lib053	SRR17380164	1,525,494	92.4	E	N/A	SOP	SOP

Table S4. Target values for 'allowable errors' (that is, disagreement between the expected and measured strain-wise abundances) for shotgun metagenomics measurements of the mock communities.

	geometric mean absolute fold differences (gmAFD)	maximum absolute fold differences (maxAFD)
DNA mock	1.12	1.41
cell mock	1.29	2.39

Table S5. Command line settings for fastp.

settings_id	cut_right_mean_quality	unqualified_percent_limit	length_required
15_100_65	15	100	65
17_100_65	17	100	65
19_100_65	19	100	65
21_100_65	21	100	65
15_100_50	15	100	50
17_100_50	17	100	50
19_100_50	19	100	50
21_100_50	21	100	50
15_40_65	15	40	65
17_40_65	17	40	65
19_40_65	19	40	65
21_40_65	21	40	65
15_40_50	15	40	50
17_40_50	17	40	50
19_40_50	19	40	50
21_40_50	21	40	50
15_60_65	15	60	65
17_60_65	17	60	65
19_60_65	19	60	65
21_60_65	21	60	65
15_60_50	15	60	50
17_60_50	17	60	50
19_60_50	19	60	50
21_60_50	21	60	50
15_80_65	15	80	65
17_80_65	17	80	65
19_80_65	19	80	65
21_80_65	21	80	65
15_80_50	15	80	50
17_80_50	17	80	50
19_80_50	19	80	50
21_80_50	21	80	50
0_100_65	0	100	65
0_100_50	0	100	50
0_40_65	0	40	65
0_40_50	0	40	50
0_60_65	0	60	65
0_60_50	0	60	50
0_80_65	0	80	65
0_80_50	0	80	50

Table S6. Summary of growth conditions for the strains in the mock communities.

species, strain	broth	atmosphere	temperature (°C)	time (hr)
A. caccae NBRC 114412	HGAM + 1% glucose + 33 mM NaOAc	anaerobic (N_2)	37	24
R. gnavus NBRC 114413	HGAM + 1% glucose + 33 mM NaOAc	anaerobic (N_2)	37	24
A. muciniphila NBRC 114322	HGAM + 1% glucose + 33 mM NaOAc	anaerobic (N_2)	37	40
F. plautii NBRC 113805	HGAM + 33 mM NaOAc	anaerobic (N_2)	37	48
B. longum NBRC 114370	HGAM + 1% glucose	anaerobic (N_2)	37	24
B. longum subsp. *longum* NBRC 114494	HGAM + 1% glucose	anaerobic (N_2)	37	24
Blautia sp. NBRC 113351	HGAM + 1% glucose	anaerobic (N_2)	37	24
C. aerofaciens NBRC 114504	HGAM + 1% glucose	anaerobic (N_2)	37	24
E. clostridioformis NBRC 113352	HGAM + 1% glucose	anaerobic (N_2)	37	24
B. uniformis NBRC 113350	HGAM + 1% glucose	anaerobic (N_2)	37	24
M. funiformis NBRC 114415	HGAM + 1% glucose + 33 mM NaOAc	anaerobic (N_2)	37	48
M. massiliensi NBRC 114414	HGAM + 1% glucose + 33 mM NaOAc	anaerobic (N_2)	37	24
P. distasonis NBRC 113806	HGAM + 1% glucose	anaerobic (N_2)	37	24
S. mutans NBRC 13955^T	HGAM + 1% glucose	anaerobic (N_2)	37	24
C. acnes subsp. *acnes* NBRC 113869	HGAM + 1% glucose	anaerobic (N_2)	37	48
L. delbrueckii subsp. *delbrueckii* NBRC 3202	HGAM + 1% glucose	anaerobic (N_2)	30	48
S. epidermidis NBRC 113846	medium 702	aerobic	30	24
E. coli NBRC 3301	medium 702	aerobic	30	24
B. subtilis subsp. *subtilis* NBRC 13719	medium 702	aerobic	30	24
P. putida NBRC 14164	medium 702	aerobic	30	24

medium 702: https://www.nite.go.jp/nbrc/catalogue/NBRCMediumDetailServlet?NO=702

Table S7. Summary of command lines.

function	tool		database, if applicable
Read processing	fastp (v0.20.0)	fastp -i {i} -I {I} -o {o} -O {O} --trim_front1 5 --trim_front2 5 --trim_tail1 1 -trim_tail2 1 --unqualified_percent_limit 100 --trim_poly_x --poly_x_min_len 10 --n_base_limit 0 --low_complexity_filter --length_required 65	
Read subsampling	seqtk (v1.3)	seqtk sample -s{seed} {reads} {depth} > {reads_depth}	
Read simulation	BBMap (v38.82)	randomreads.sh ref={ref} out1={out1} out2={out1} reads={reads} adderrors=f paired=t minlength={minlength} maxlength={maxlength}	
Quantification	kalllisto (v0.46.1)	kallisto index -i {index} {ref.fna}<br>kallisto quant --plaintext -i {db} -o {o} {read1} {read2}<br>cat {o}/abundance.tsv > {report}	reference genome sequences, indexed using kallisto's index command
Taxonomic profiling	mOTUs2 (v2.6.1)	motus map_tax -b -f {read1} -r {read2} -o {map}<br>motus calc_mgc -i {map} -o {calc}<br>motus calc_motu -k mOTU -q -i {calc}-o {motus2.report }	default database (db_mOTU_v2.6.1)
	MetaPhlAn3 (v3.0.9)	cat {read1} {read2} > {read12}<br><br>bowtie2 -x {db} --very-sensitive --no-unal -U {read12} -S {map}<br>samtools view -q 5 {map} > {map_q5}<br>metaphlan {map_q5} --input_type sam -o {metaphlan3.report }	default database (mpa_v30_CHOCOPhlAn_201901)
	kraken2 (v2.1.1)	kraken2 --confidence 0.05 --db={db} --report {kraken2.report} --output {map} --paired {read1} {read2}<br>kreport2mpa.py -r {kraken2.report } -o {kraken2.report mpa} --read_count --no-intermediate-ranks	GTDB_release95<br>k2_standard_20210517
	bracken (v2.6.0)	bracken -d {db} -i {kraken2.report} -r {read_length} -l S -t 1000 -w {bracken.report}<br>kreport2mpa.py -r {bracken.report} -o {bracken.report.mpa} --read_count --no-intermediate-ranks	GTDB_release95<br>k2_standard_20210517

Sources of databases for MetaPhlAn3, Kraken2 and Bracken:
http://cmprod1.cibio.unitn.it/biobakery3/metaphlan_databases/mpa_v30_CHOCOPhlAn_201901.tar
https://genome-idx.s3.amazonaws.com/kraken/k2_standard_20210517.tar.gz
http://ftp.tue.mpg.de/ebio/projects/struo2/GTDB_release95/kraken2/hash.k2d
http://ftp.tue.mpg.de/ebio/projects/struo2/GTDB_release95/kraken2/taxo.k2d
http://ftp.tue.mpg.de/ebio/projects/struo2/GTDB_release95/kraken2/opts.k2d
http://ftp.tue.mpg.de/ebio/projects/struo2/GTDB_release95/bracken/database100mers.kmer_distrib
http://ftp.tue.mpg.de/ebio/projects/struo2/GTDB_release95/bracken/database100mers.kraken
http://ftp.tue.mpg.de/ebio/projects/struo2/GTDB_release95/bracken/database150mers.kmer_distrib
http://ftp.tue.mpg.de/ebio/projects/struo2/GTDB_release95/bracken/database150mers.kraken

Supplementary Figures

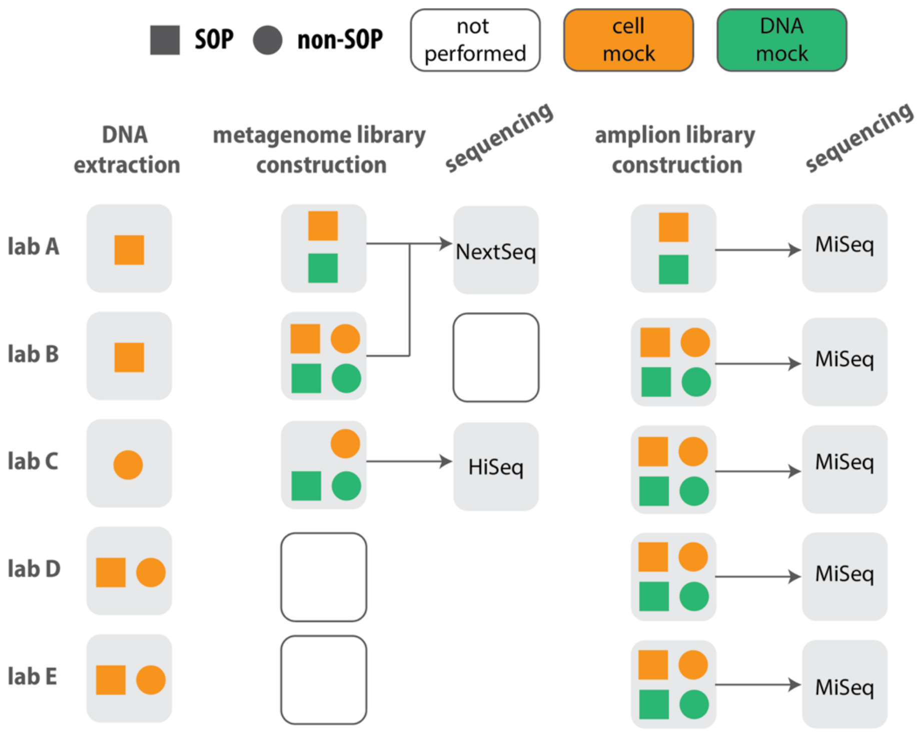

Figure S1. Schematic of the collaborative study for characterization and demonstration of fit-for-purpose of the newly developed DNA and cell mock communities. Detailed descriptions of the SOPs and non-SOPs are provided in the main and supplementary methods and an overview of all samples and associated sequencing data is provided in Table S3.

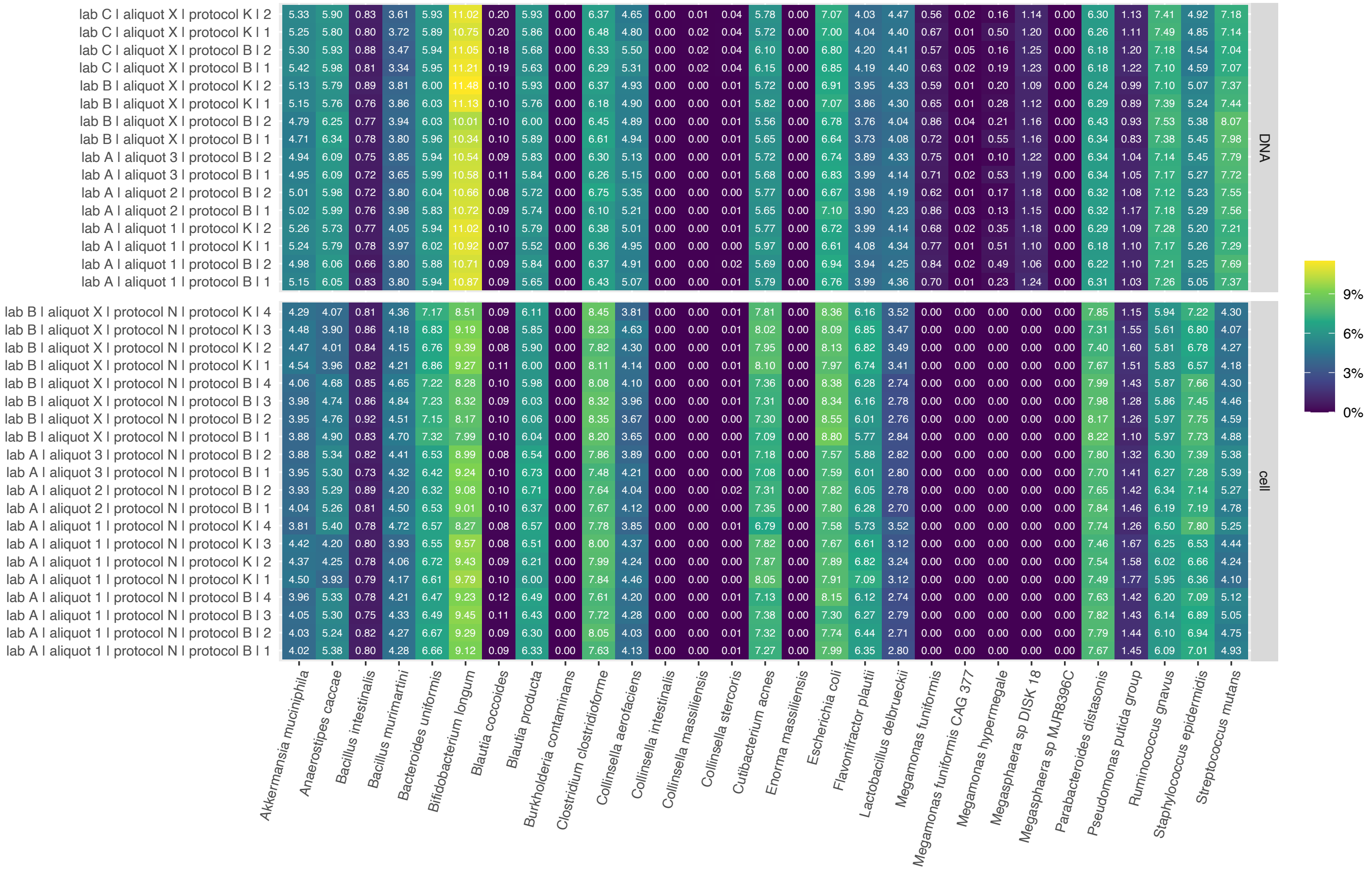

Figure S2. Taxonomic profiles of individual replicates for the DNA mock community (*n* = 16, upper facet) and cell mock community (*n* = 20, lower facet), generated by MetaPhlAn3. Shotgun metagenome libraries were prepared by two or three laboratories following SOPs for DNA extraction (protocol N) and sequencing library construction (protocols B and K), as indicated in the sample keys on the *y* axis. Fill colors and corresponding values overlayed onto the heatmap reflect relative abundances, as percentages.

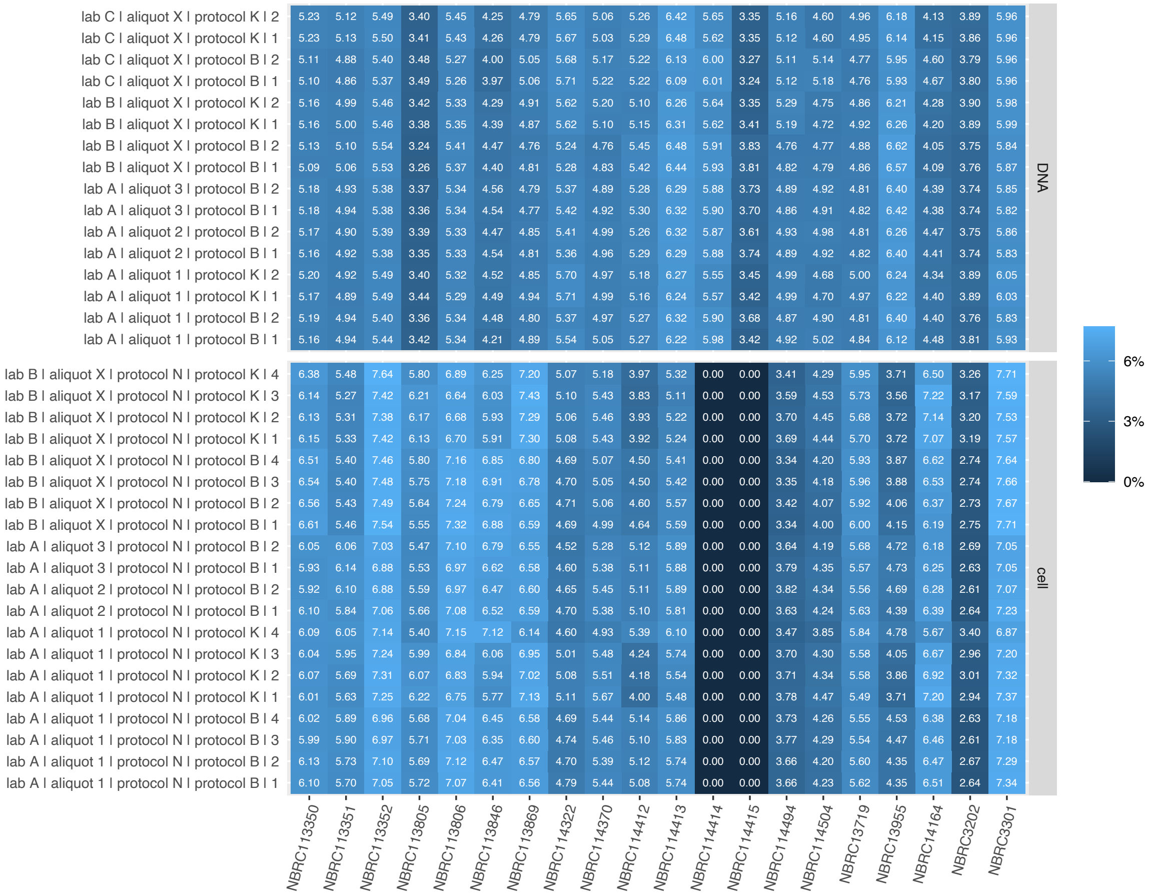

Figure S3. Abundances of each strain in the mock communities as determined by shotgun metagenomics and quantification by kallisto. The top and bottom panel show results for the DNA and cell mock community, respectively, as indicated in the facet labels. Shotgun metagenomics libraries were prepared by two or three laboratories following SOPs for DNA extraction (protocol N) and sequencing library construction (protocols B and K), as indicated in the sample keys on the *y* axis. Fill colors and corresponding values overlayed onto the heatmap reflect relative abundances, as percentages. Note that strains NBRC 114414 and NBRC 114415 are not part of the cell mock community.

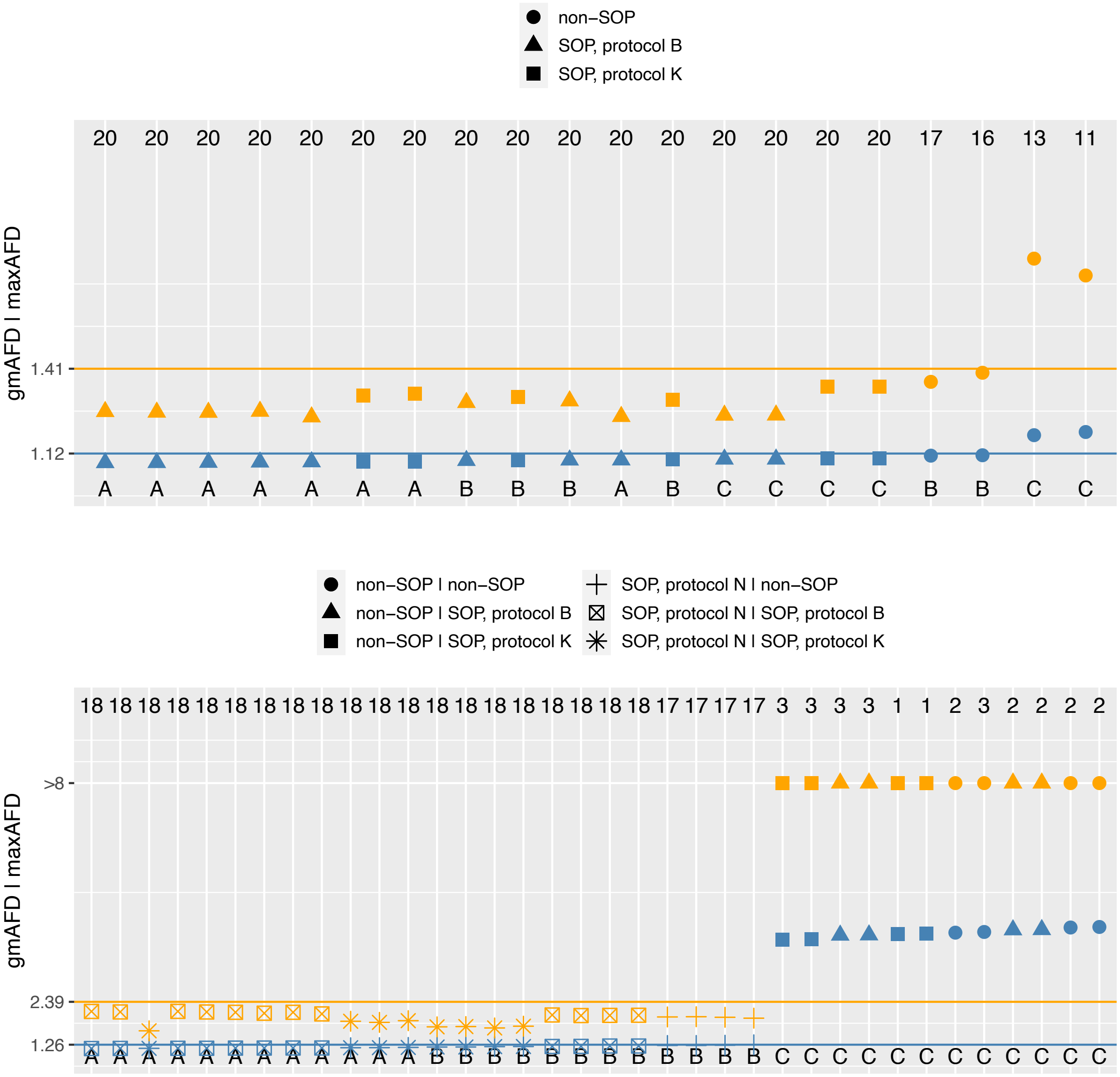

Figure S4. Application of guidelines values for assessing methods for libray construction (DNA mock community, top panel) and DNA extraction + libray construction (cell mock community, lower panel). Blue and orange symbols represent the geometric mean and maximum of strain-wise absolute fold differences, respectively; corresponding “acceptance” values (see Table S4) are shown as horizontal lines with the same colors. Letters indicate participating laboratories. Numbers plotted at the top show the number of strains satisfying the “acceptance” range for strain-wise abundances (see Fig.1C).

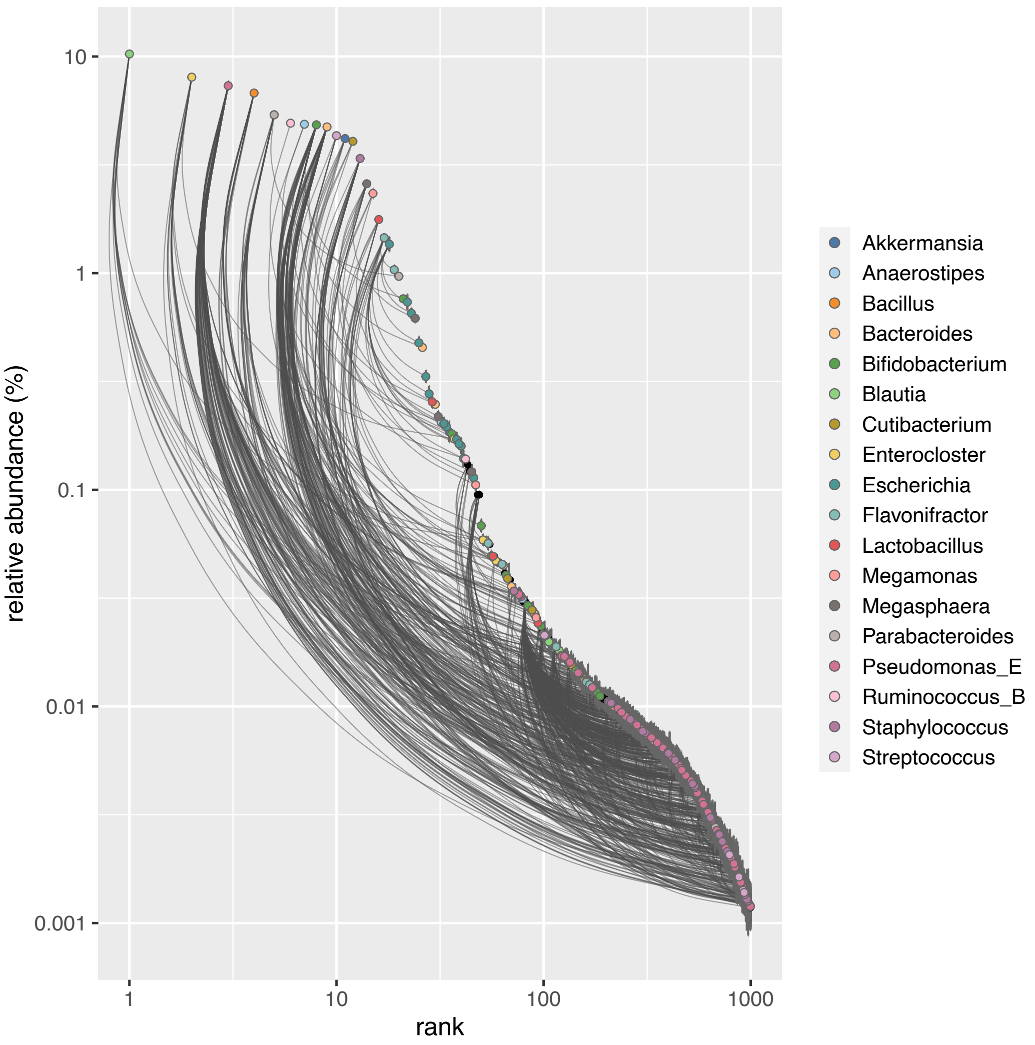

Figure S5. Rank abundance curve of species detected by kraken2 using the GTDB database (that is, kraken2_gtdb). Note that only the top-1000 most abundant species (based on the geometric mean) are shown. Data are shown as the geometric mean (circles) and standard deviation (error bars) of 16 measurements for the DNA mock community, performed by three laboratories following SOPs for DNA extraction and sequencing library construction. Grey curved segments connect species belonging to the same genus, originating from the most abundant species for a given genus.

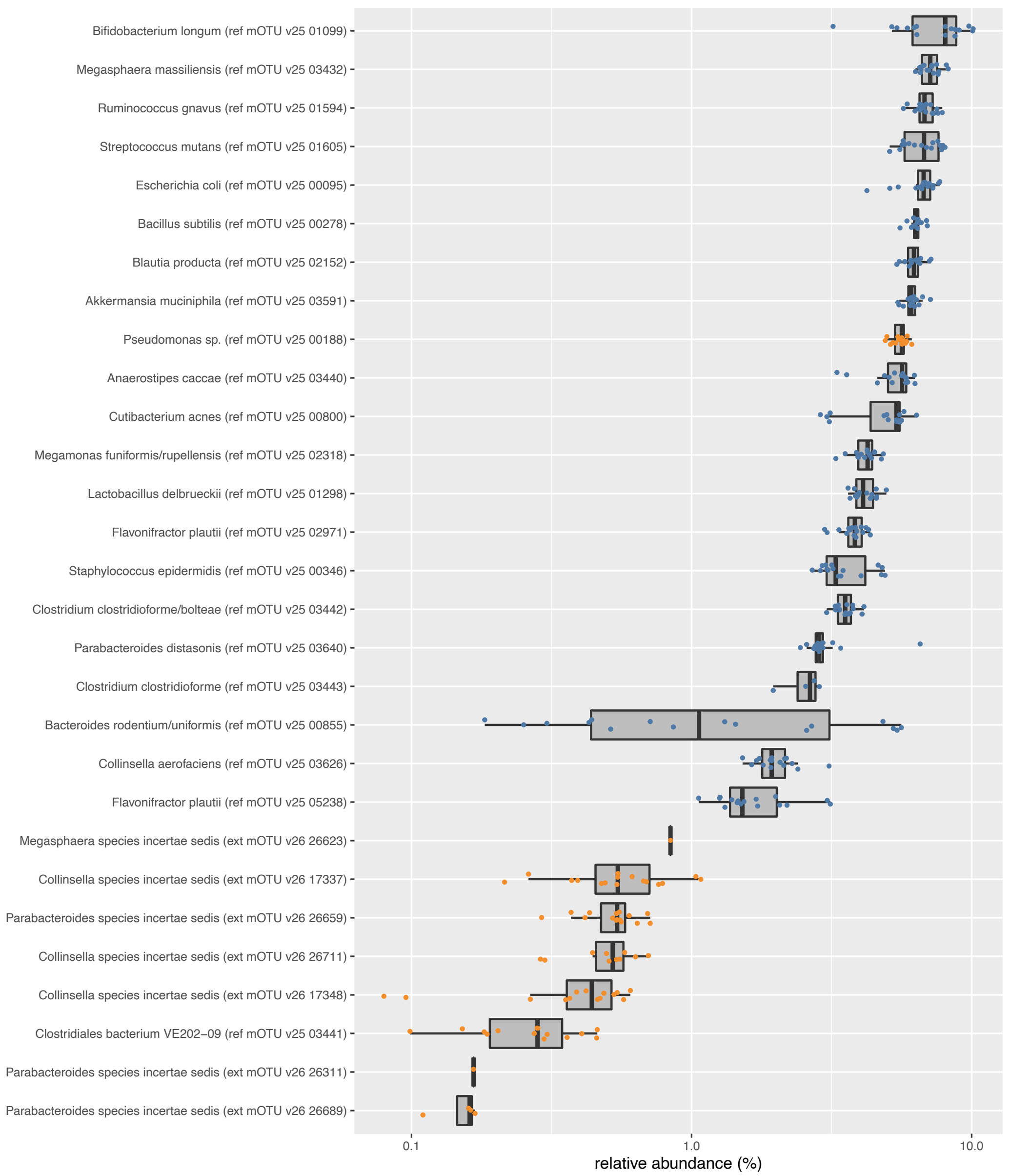

Figure S6. Species profiles for the DNA mock community as determined by mOTUs2 for 16 replicated measurements of the DNA mock community, performed by three laboratories following SOPs for DNA extraction and sequencing library construction. Data are shown as boxplots with individual datapoints overlayed. Only species with an mean abundance of at least 0.01% across replicates are shown. Blue and orange circles show "expected" and "unexpected" species, respectively, based on string matches to the species names. For the boxplots, the tick vertical line represents the median, hinges show the 25th and 75th percentiles, whiskers extend to the largest and smallest value at most 1.5× the IQR (interquartile range) from the upper and lower hinges, respectively. Individual datapoints are overlayed with jitter.

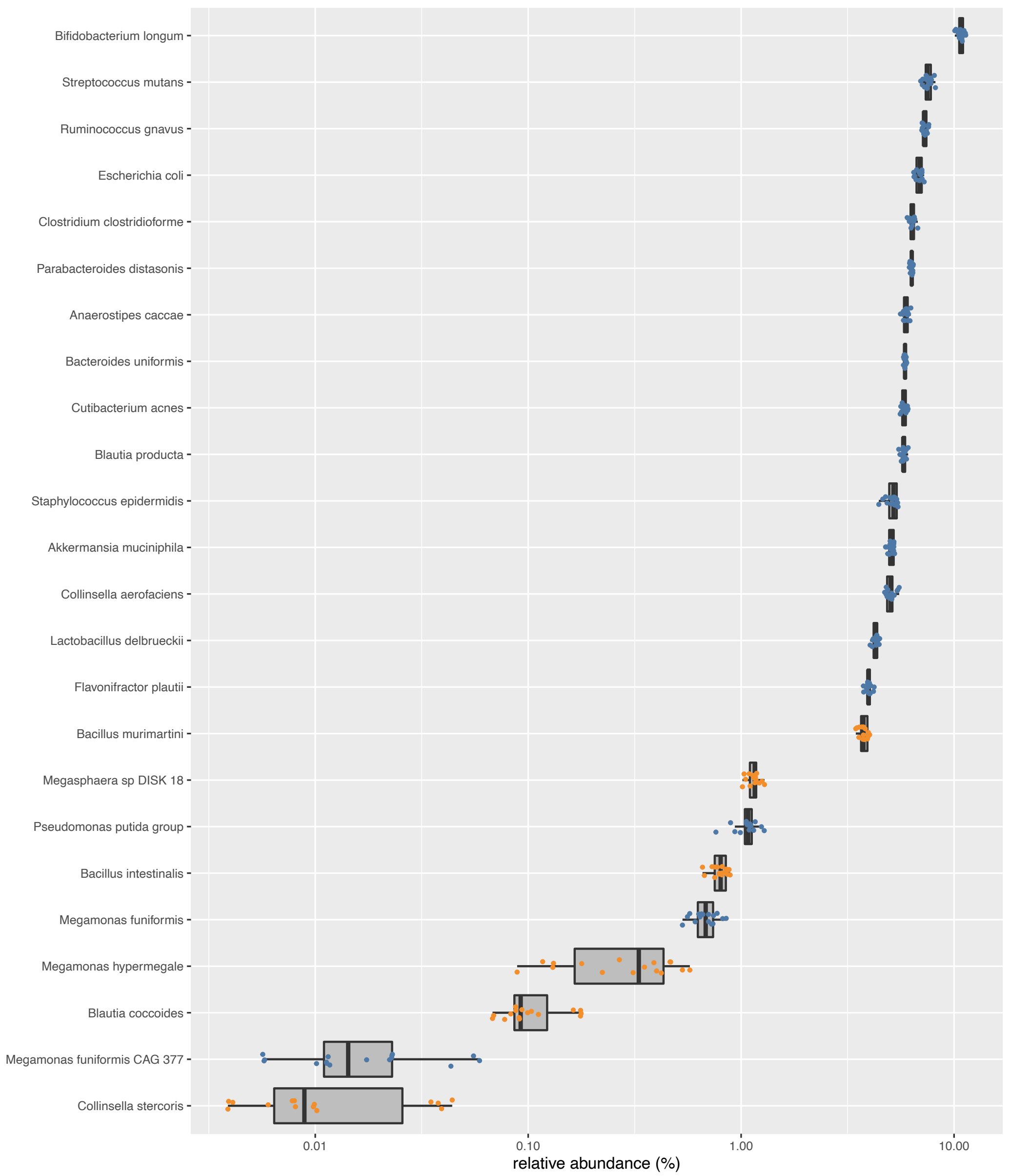

Figure S6, cont'd. Same for MetaPhlAn3.

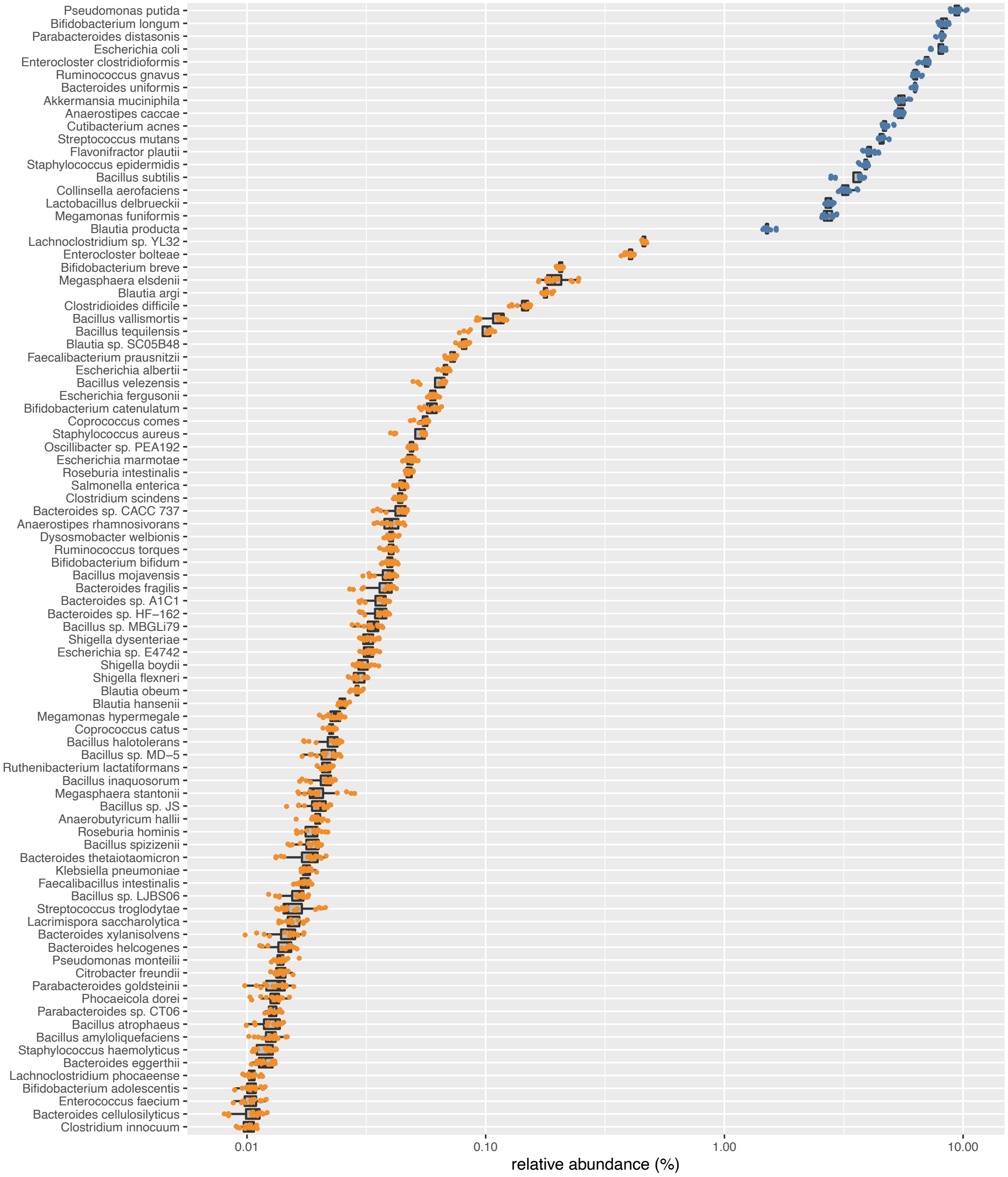

Figure S6, cont'd. Same for kraken2_refseq.

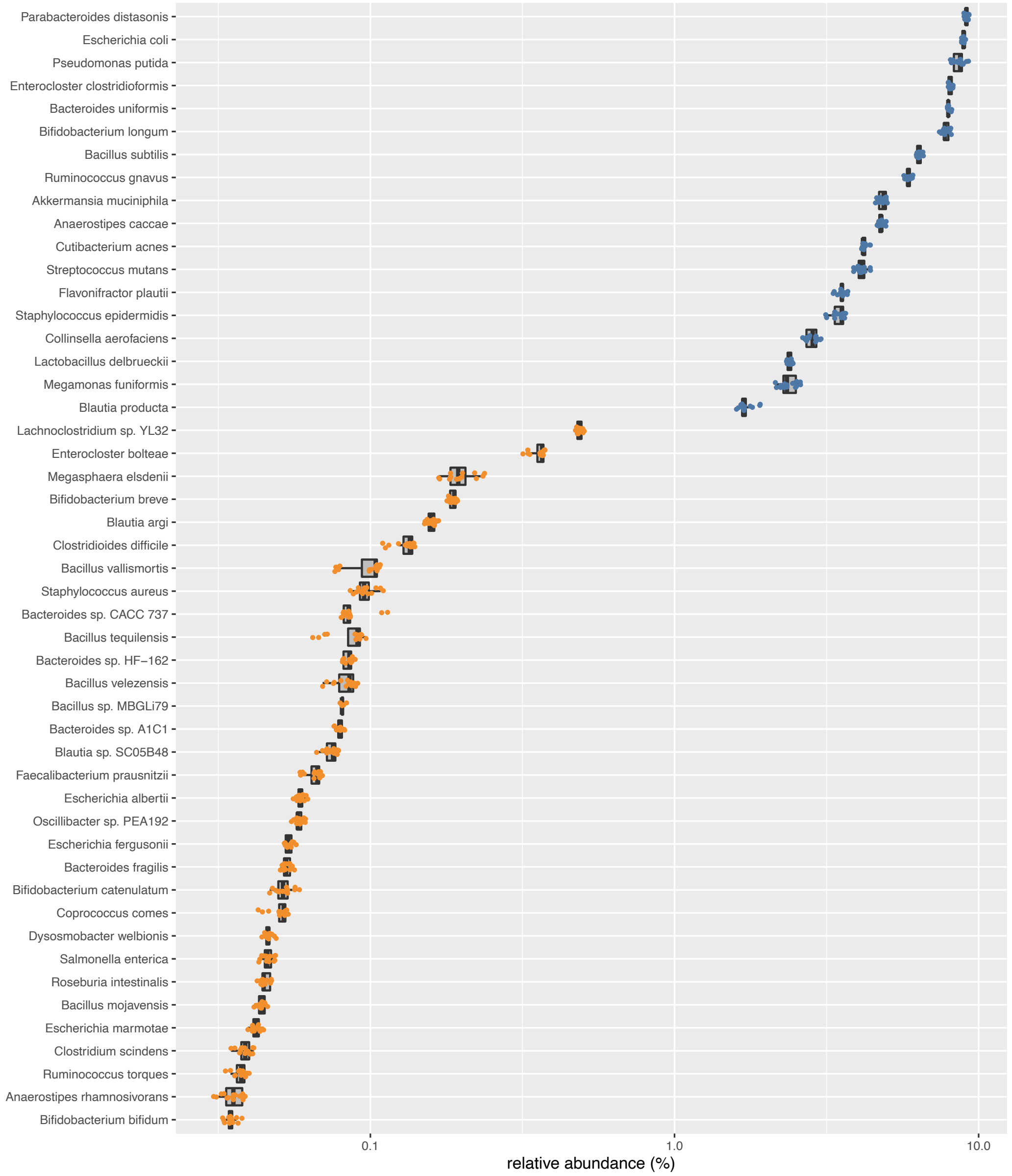

Figure S6, cont'd. Same for bracken_refseq.

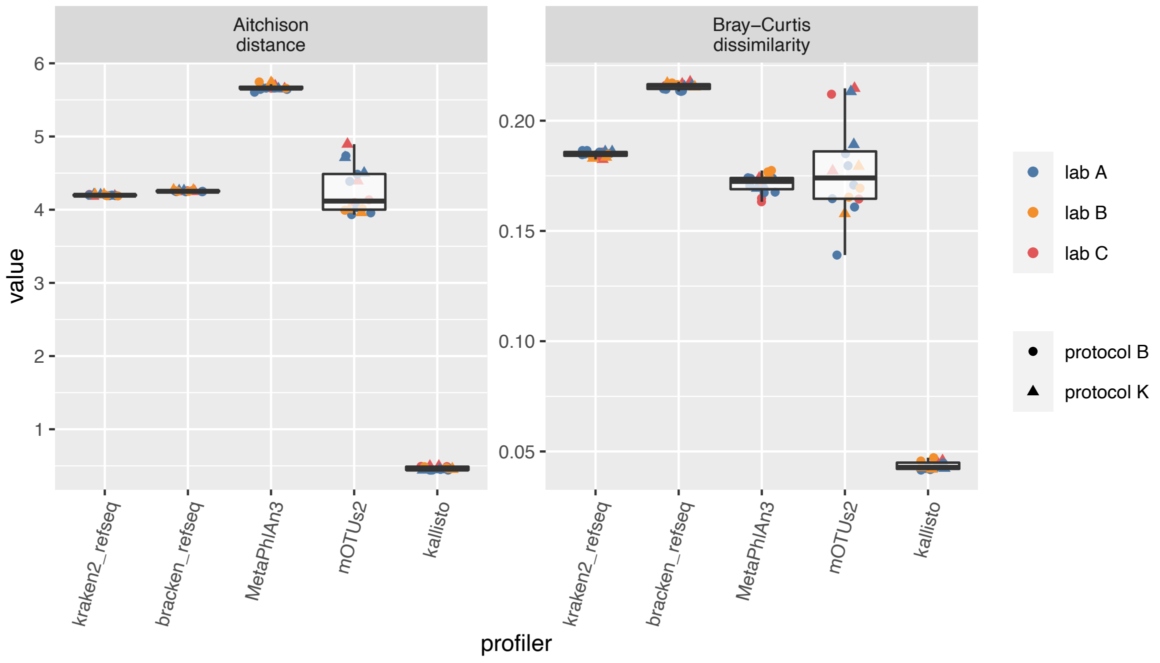

Figure S7. Agreement between the expected species profiles (relative abundances) and profiles generated by different taxonomic profilers. Agreement was calculated as Aitchison distances or Bray-Curtis dissimilarities between measured and expected species profiles. Note that only subcompositions of expected species (based on string matches to the assigned taxonomy strings) were considered, with rescaling to 100% for calculation of Bray-Curtis dissimilarities and replacement of zeros with 0.1% for calculation of Aitchison distances. Data are calculated for 16 individual measurements of the DNA mock community as performed using the SOPs (shapes) in three laboratories (colors). For the boxplots, the tick horizontal line represents the median, hinges show the 25th and 75th percentiles, whiskers extend to the largest and smallest value at most 1.5× the IQR (interquartile range) from the upper and lower hinges, respectively. Individual datapoints are overlayed with jitter.

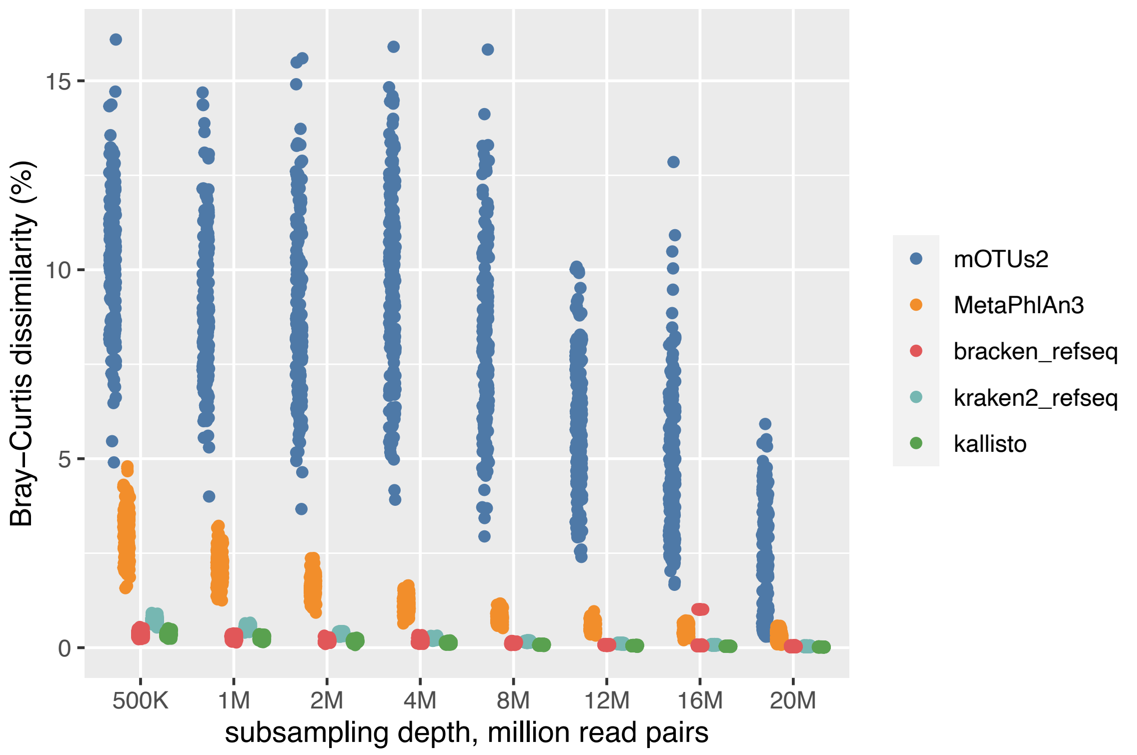

Figure S8. Bray-Curtis dissimilarities between randomly subsampled datasets as generated using different profilers. To this end, we randomly subsampled four deeply sequenced HiSeq libraries, for the DNA mock community generated using protocols B and K (SRR15195689, SRR15195686, SRR15195688 and SRR15195685 in Table S3), to varying sequencing depths (0.5 to 20 million read pairs per sample). Subsequently, for each library and subsampling depth, all possible (45 per library, or 180 total) pairwise distances/dissimilarities between 10 random subsamples for each subsampling depth, as plotted on the *x* axis. For Bray-Curtis dissimilaties, all identified species, including false positives, were considered. For Aitchison distances, only expected species were considered.

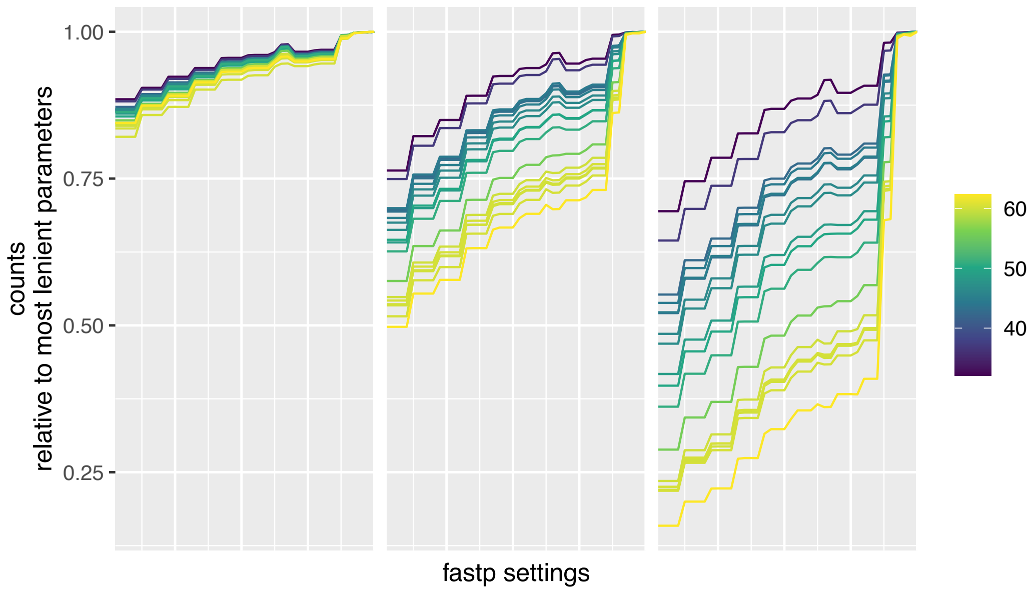

Figure S9. Effect of read quality trimming and filtering on the proportion of retained reads. Data for three representative libraries with high (left; library metagenome_mockCell_labA_lib038 in Table S3, Q30 bases of 90.1%), medium (middle; library metagenome_mockCell_labB_lib043, Q30 bases of 78.2%) and low (right; library metagenome_mockCell_labB_lib039, Q30 bases of 71.7%) raw base quality are shown. Values shown on the *y* axis represent the ratio of retained reads for a given fastp setting (*x* axis) to the counts observed for the most lenient settings for trimming and filtering (that is, settings 0_100_50 in Table S5). Each line represents a different strain, with colors indicating the genomic GC content (%) as indicated in the legend.

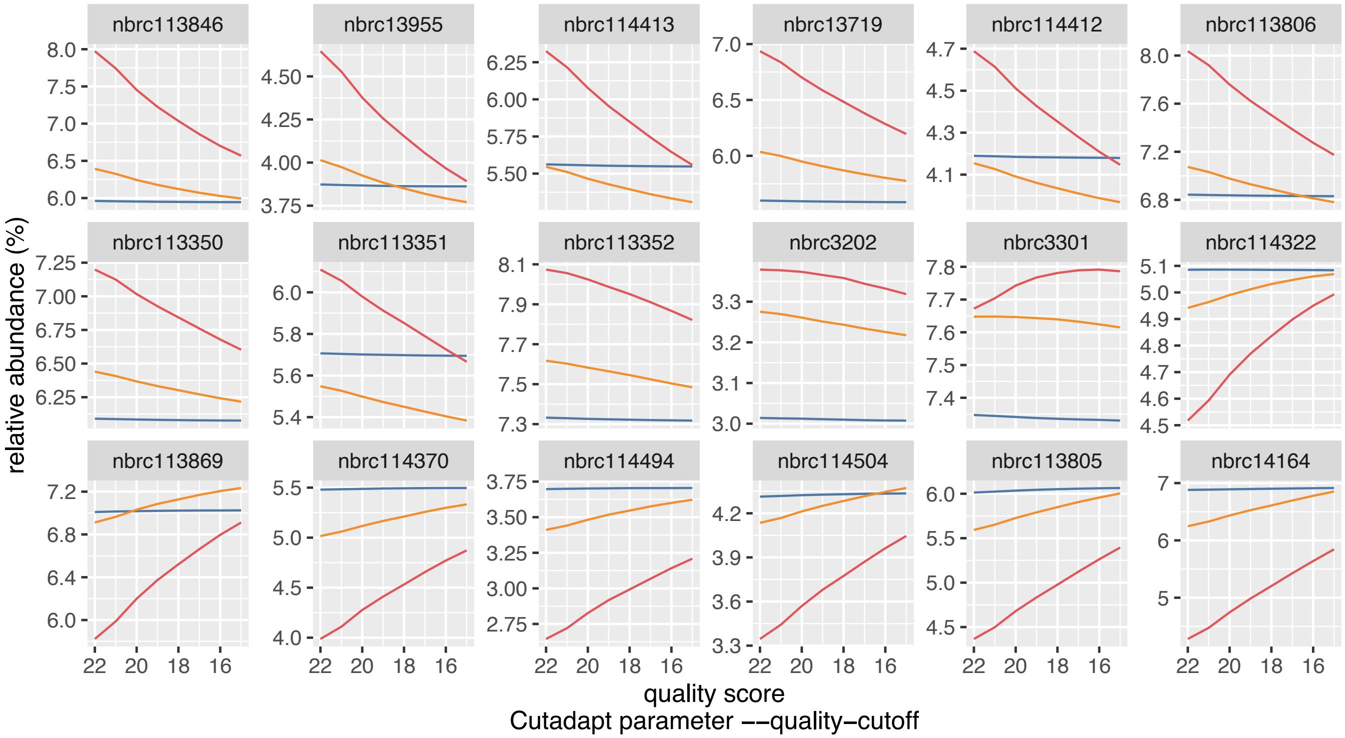

Figure S10. Effect of read trimming and filtering using Cutadapt on strain-wise abundances. To this end, we subjected reads from three representative libraries with varying raw base call to initial trimming using fastp (settings 0_100_50 in Table S5) and then performed further quality trimming using Cutadapt for a range of quality thresholds as depicted on the *x* axis. Libraries are as in Figure S9, namely library metagenome_mockCell_labA_lib038 (Q30 bases of 90.1%, shown in blue), library metagenome_mockCell_labB_lib043 (Q30 bases of 78.2%, shown in orange) and library metagenome_mockCell_labB_lib039 (Q30 bases of 71.7%, shown in red). Facets (strains) are sorted by increasing genomic GC content.

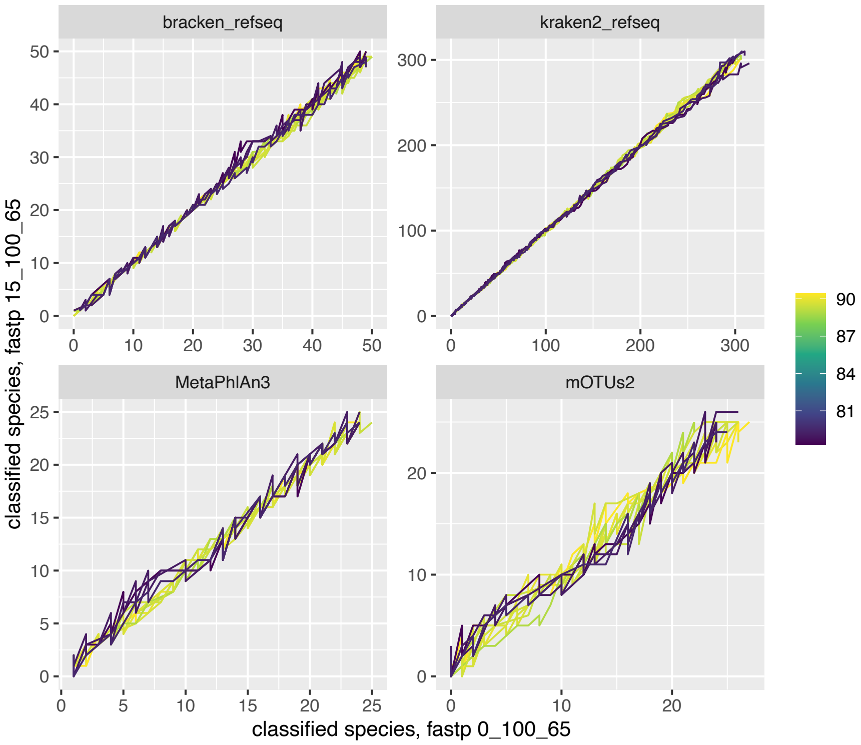

Figure S11. Effect of quality trimming on number of classified species. For each profiler, the number of classified species was calculated for decreasing abundance thresholds as in Figure 3A for two different fastp settings. Plots represent scatter plots of the number of classified species for each abundance threshold for datasets with quality trimming (*x* axis) and without quality trimming (*y* axis). Lines are colored according to the percentage of bases with quality scores of >30 as indicated in the legend.

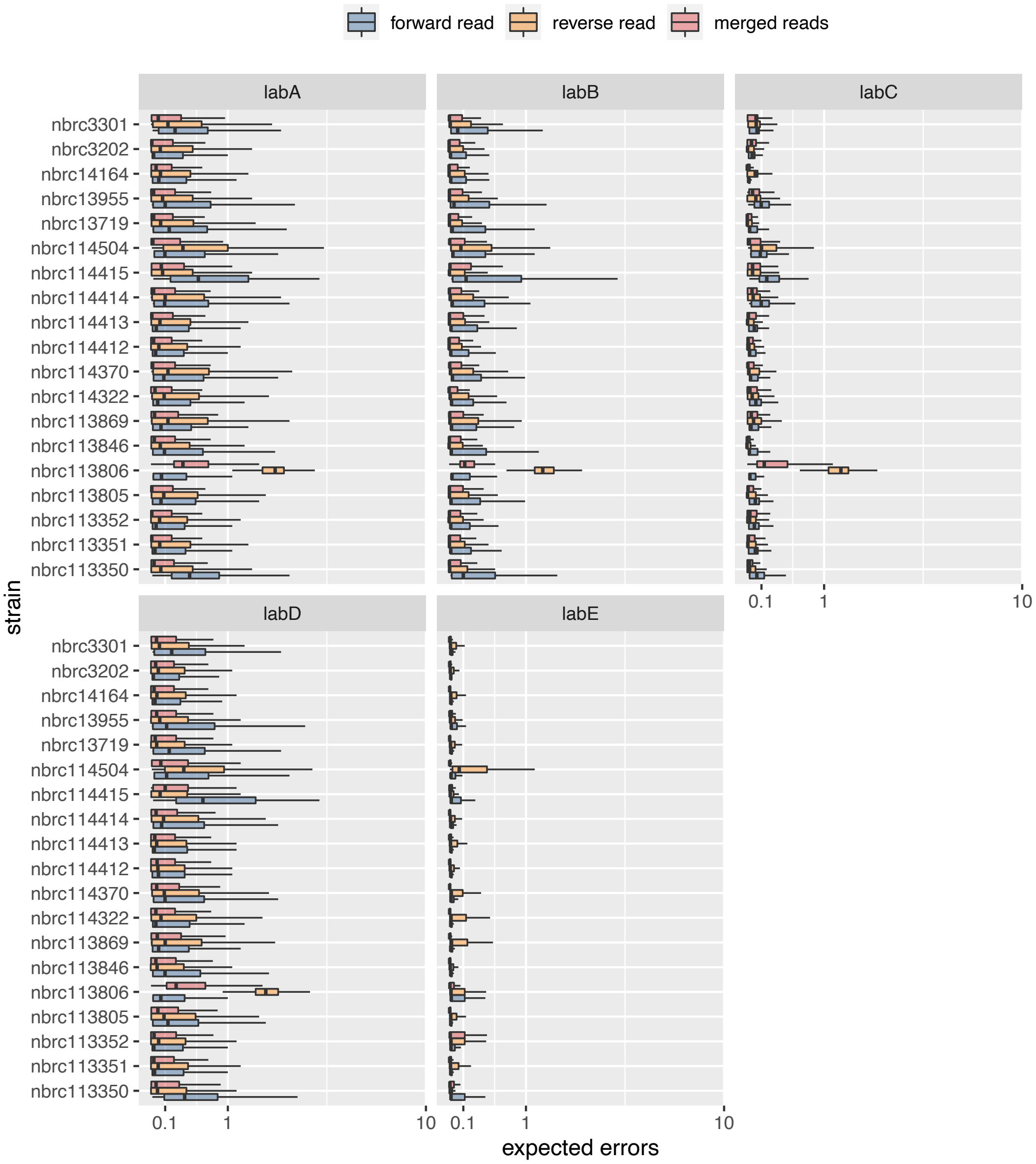

Figure S12. Distribution of strain-wise expected errors (EE), as calculated using USERACH's fastq_filter command, for the amplicon sequencing data; all available data for the DNA mock community (see Table S3) were included. For the boxplots, the tick horizontal line represents the median, hinges show the 25th and 75th percentiles, whiskers extend to the largest and smallest value at most 1.5× the IQR (interquartile range) from the upper and lower hinges, respectively; outliers are not shown. Note that participants A through D employed V2 chemistry for sequencing on a MiSeq instrument and participant E used V3 chemistry.

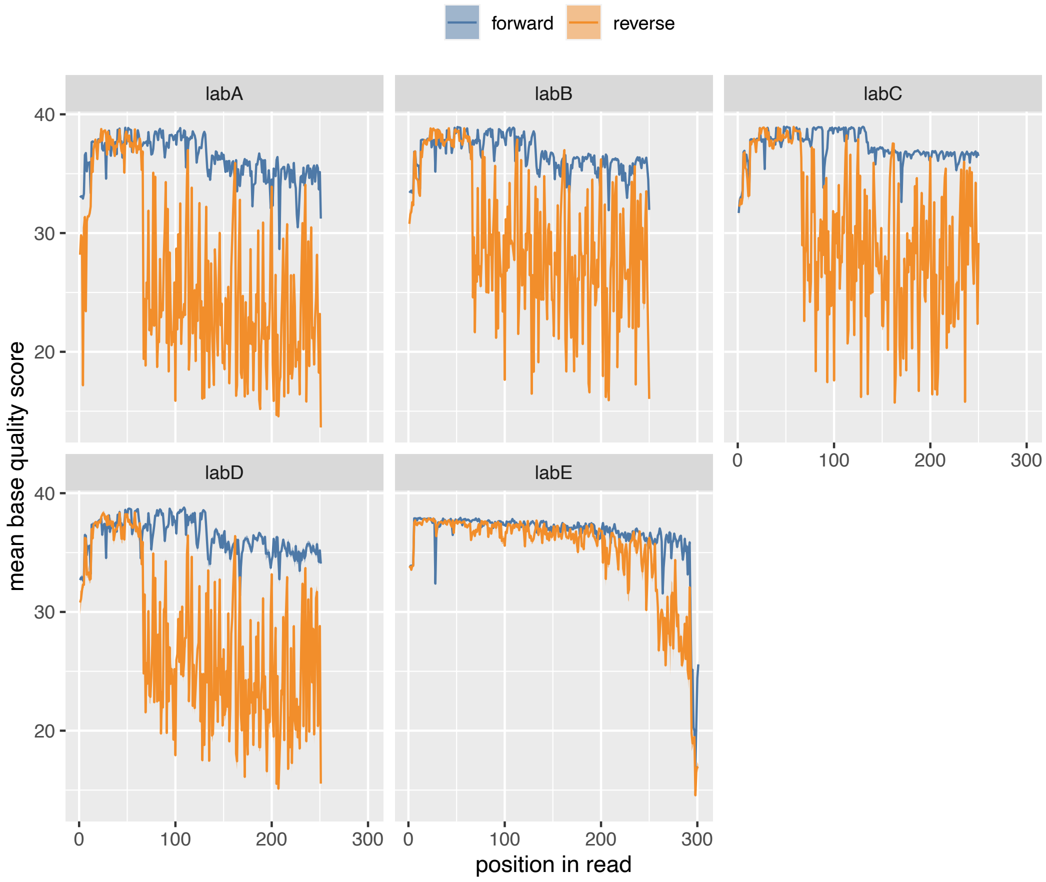

Figure S13. Raw base quality scores for sequences assigned to *P. distasonis* NBRC 113806. Data are shown as the mean (solid lines) and standard deviation (ribbons, if visible) of all available data for the DNA mock community (see Table S3). Note that participants A through D employed V2 chemistry for sequencing on a MiSeq instrument and participant E used V3 chemistry.
